# A deterministic equation to predict the accuracy of multi-population genomic prediction with multiple genomic relationship matrices

**DOI:** 10.1186/s12711-020-00540-y

**Published:** 2020-04-28

**Authors:** Biaty Raymond, Yvonne C. J. Wientjes, Aniek C. Bouwman, Chris Schrooten, Roel F. Veerkamp

**Affiliations:** 1 Animal Breeding and Genomics, Wageningen University and Research, P.O. Box 338, 6700 AH Wageningen, The Netherlands; 2 Biometris, Wageningen University and Research, 6700AA Wageningen, The Netherlands; 3 CRV BV, P.O. Box 454, 6800 AL Arnhem, The Netherlands

## Abstract

**Background:**

A multi-population genomic prediction (GP) model in which important pre-selected single nucleotide polymorphisms (SNPs) are differentially weighted (MPMG) has been shown to result in better prediction accuracy than a multi-population, single genomic relationship matrix (${\mathbf{GRM}}$) GP model (MPSG) in which all SNPs are weighted equally. Our objective was to underpin theoretically the advantages and limits of the MPMG model over the MPSG model, by deriving and validating a deterministic prediction equation for its accuracy.

**Methods:**

Using selection index theory, we derived an equation to predict the accuracy of estimated total genomic values of selection candidates from population $A$ ($r_{{{\mathbf{EGV}}_{{A_{T} }} }}$), when individuals from two populations, $A$ and $B$, are combined in the training population and two ${\mathbf{GRM}}$, made respectively from pre-selected and remaining SNPs, are fitted simultaneously in MPMG. We used simulations to validate the prediction equation in scenarios that differed in the level of genetic correlation between populations, heritability, and proportion of genetic variance explained by the pre-selected SNPs. Empirical accuracy of the MPMG model in each scenario was calculated and compared to the predicted accuracy from the equation.

**Results:**

In general, the derived prediction equation resulted in accurate predictions of $r_{{{\mathbf{EGV}}_{{A_{T} }} }}$ for the scenarios evaluated. Using the prediction equation, we showed that an important advantage of the MPMG model over the MPSG model is its ability to benefit from the small number of independent chromosome segments ($M_{e}$) due to the pre-selected SNPs, both within and across populations, whereas for the MPSG model, there is only a single value for $M_{e}$, calculated based on all SNPs, which is very large. However, this advantage is dependent on the pre-selected SNPs that explain some proportion of the total genetic variance for the trait.

**Conclusions:**

We developed an equation that gives insight into why, and under which conditions the MPMG outperforms the MPSG model for GP. The equation can be used as a deterministic tool to assess the potential benefit of combining information from different populations, e.g., different breeds or lines for GP in livestock or plants, or different groups of people based on their ethnic background for prediction of disease risk scores.

## Background

Genomic prediction (GP) [1] has become widely accepted and has been successfully implemented in both animal and plant breeding schemes [2–5]. However, for accurate GP it is essential that the training population is large [6–8]. For numerically small populations, e.g., numerically small breeds or lines in livestock or numerically small human ethnic groups, it is difficult or impossible to assemble a large enough training population that can accurately predict the genomic values. Therefore, the accuracy of GP in numerically small populations is limited [9].

A potential option to increase the accuracy of GP in numerically small populations is to use a large training population made up of individuals from multiple populations, including the target population, a method known as multi-population GP. Results from dairy cattle indicate that this approach can lead to substantial increases in the accuracy of GP for numerically small breeds, if the training population is made up of individuals from different but closely-related breeds that have recently had substantial exchanges of genetic material, and that a large number of individuals from the additional breed is included [10]. However, in cases in which distantly related breeds were combined in a single training population, increases in the accuracy of multi-population GP were limited compared to that of within-population GP [11–13].

Different statistical approaches have been adopted for multi-population GP. One approach, and the most straightforward, is the univariate single-trait approach in which individuals from multiple populations are pooled and treated as individuals from the same population in a training population. The underlying assumption of this approach is that the genetic correlation between the populations is equal to 1. Deviations from this assumption, for example, when distantly related populations are combined, can result in prediction accuracies that are even lower than the accuracy of within-population GP [11]. Another approach used for multi-population GP is to consider phenotypes of individuals from different populations, e.g. phenotypes from different, but correlated traits [14, 15]. The advantage of this multi-trait approach is that it can consider that the genetic correlation between populations can be less than 1. In the worst-case scenario, the accuracy of multi-population GP using a multi-trait approach is expected to be the same as the accuracy of within-population GP, but not lower [16].

In both the single-trait and the multi-trait approaches for multi-population GP, DNA markers such as single nucleotide polymorphisms (SNPs) are equally weighted in the model. However, some studies have shown that accuracy of GP can be improved by prioritising certain SNPs that have a significant effect on the trait or by incorporating prior biological knowledge on SNPs in the prediction model [17–19]. Based on that rationale, and to improve the potential to use information from other populations, Raymond et al. [20] proposed the so-called multi-breed, multiple genomic relationship matrices (GRM) GP model (MBMG), which in this study is generalised as MPMG, given that the model can be applied in other species, e.g., plant or humans. The three key features of this model are: (1) SNPs are pre-selected based on prior knowledge of potential causal effects and are used to build a ${\mathbf{GRM}}$; (2) the remaining unselected SNPs are used to make a separate ${\mathbf{GRM}}$, in order to explain the residual genetic variance for the trait; and (3) information of each population in the training population is weighed by their genetic correlation with the other populations and with the selection candidates. The MPMG model is equivalent to a model with a single GRM, in which different weights are applied to the two classes of SNPs. Using both real and simulated data, Raymond et al. [20] showed that the MPMG model can result in significant increases in the accuracy of GP, as compared with a multi-trait approach in which all SNPs are pooled together in a single ${\mathbf{GRM}}$ (MPSG) [20]. Given the superior performance of the MPMG model over MPSG, the objective of this study was to underpin theoretically the advantages and limits of the MPMG model as compared to the MPSG model, by deriving and validating a deterministic prediction equation for the accuracy.

## Methods

### Multi-population, multiple genomic relationship matrices (MPMG) model

We assume that individuals from populations $A$ and $B$ are combined in the training population to predict the genomic value of selection candidates from population $A$ using the MPMG model following Raymond et al. [20]. The MPMG model is a bivariate model that considers the phenotypes of individuals from populations $A$ and $B$ for the same trait as those from two different, but correlated traits. The prior biological knowledge that exists about the effect sizes of the SNPs is used to pre-select important SNPs that are used to build one ${\mathbf{GRM}}$. The remaining SNPs are used to build a second ${\mathbf{GRM}}$ to explain the residual genetic variance not explained by the pre-selected SNPs. Both ${\mathbf{GRM}}$ are fitted simultaneously in the bivariate model [20]. The model can be specified as:

1 $$\left[\begin{array}{c}{\mathbf{y}}_{A}\\ {\mathbf{y}}_{B}\end{array}\right]=\left[\begin{array}{cc}{\mathbf{1}}& {\mathbf{0}}\\ {\mathbf{0}}& {\mathbf{1}}\end{array}\right]\left[\begin{array}{c}{\mu }_{A}\\ {\mu }_{B}\end{array}\right]+\left[\begin{array}{cc}{\mathbf{W}}_{{1}_{A}}& {\mathbf{0}}\\ {\mathbf{0}}& {\mathbf{W}}_{{1}_{B}}\end{array}\right]\left[\begin{array}{c}{\mathbf{E}\mathbf{G}\mathbf{V}}_{{1}_{A}}\\ {\mathbf{E}\mathbf{G}\mathbf{V}}_{{1}_{B}}\end{array}\right]+\left[\begin{array}{cc}{\mathbf{W}}_{{2}_{A}}& {\mathbf{0}}\\ {\mathbf{0}}& {\mathbf{W}}_{{2}_{B}}\end{array}\right]\left[\begin{array}{c}{\mathbf{E}\mathbf{G}\mathbf{V}}_{{2}_{A}}\\ {\mathbf{E}\mathbf{G}\mathbf{V}}_{{2}_{B}}\end{array}\right]+\left[\begin{array}{c}{\mathbf{e}}_{A}\\ {\mathbf{e}}_{B}\end{array}\right]$$

where subscripts 1 and 2 represent the first and second ${\mathbf{GRM}}$ fitted in the model and subscripts $A$ and $B$ represent the populations $A$ and $B$. ${\mathbf{y}}_{A}$ is a vector of phenotypes for individuals from population $A$ and ${\mathbf{y}}_{B}$ is a vector of phenotypes for individuals from population $B$, $\mu$ is the trait mean, ${\mathbf{W}}_{1}$ and ${\mathbf{W}}_{2}$ are incidence matrices linking phenotypes to the two estimated genomic values,${\mathbf{EGV}}_{1}$ and ${\mathbf{EGV}}_{2}$, and ${\mathbf{e}}$ is the residual. Estimated genomic values are assumed to be normally distributed as:

$$\left[ {\begin{array}{*{20}c} {{\mathbf{EGV}}_{{1_{A} }} } \\ {{\mathbf{EGV}}_{{1_{B} }} } \\ \end{array} } \right]\sim N\left( {0,{\mathbf{K}}_{1} \otimes {\mathbf{GRM}}_{1} } \right),$$

$${\text{and}}\;\;\left[ {\begin{array}{*{20}c} {{\mathbf{EGV}}_{{2_{A} }} } \\ {{\mathbf{EGV}}_{{2_{B} }} } \\ \end{array} } \right]\sim N\left( {0,{\mathbf{K}}_{2} \otimes {\mathbf{GRM}}_{2} } \right),$$

with $${\mathbf{K}}_{1} = \left[ {\begin{array}{*{20}c} {\sigma_{{g_{{A_{1} }} }}^{2} } & {\sigma_{{g_{{A,B_{1} }} }} } \\ {\sigma_{{g_{{A,B_{1} }} }} } & {\sigma_{{g_{{B_{1} }} }}^{2} } \\ \end{array} } \right]$$ and $${\mathbf{K}}_{2} = \left[ {\begin{array}{*{20}c} {\sigma_{{g_{{A_{2} }} }}^{2} } & {\sigma_{{g_{{A,B_{2} }} }} } \\ {\sigma_{{g_{{A,B_{2} }} }} } & {\sigma_{{g_{{B_{2} }} }}^{2} } \\ \end{array} } \right]$$, where $\sigma_{{g_{A} }}^{2}$ and $\sigma_{{g_{B} }}^{2}$ are genetic variances in populations $A$ and $B$, respectively, and $\sigma_{{g_{A,B} }}$ is the genetic covariance between the populations. The multi-population ${\mathbf{GRM}}$ fitted in the MPMG model are calculated according to Wientjes et al. [21].

### Theory

In the following derivation, the main interest is to predict the accuracy of the estimated total genomic value of selection candidates from population $A$ ($r_{{{\mathbf{EGV}}_{{A_{T} }} }}$). $r_{{{\mathbf{EGV}}_{{A_{T} }} }}$ is a product of the accuracy of estimating SNP effects ($r_{SNP}$) and the square root of the proportion of total genetic variance explained by SNPs ($\rho$) [6, 22, 23]. As a foundation, we will first derive an equation to predict $r_{{{\mathbf{EGV}}_{{A_{T} }} }}$ for within-population GP using two ${\mathbf{GRM}}$, made from two separate sets of SNPs, that are fitted simultaneously in a GREML model. Subsequently, we will derive the full equation to predict $r_{{{\mathbf{EGV}}_{{A_{T} }} }}$ when individuals from two populations, $A$ and $B$, are combined in the training population and two separate ${\mathbf{GRM}}$ are fitted simultaneously in a GREML model (MPMG). For the derivations, we will use selection index theory [24], and build upon works from Daetwyler et al. [6] and Wientjes et al. [16], who have done similar derivations for within- and multi-population GP models in which all SNPs are equally weighted in a single ${\mathbf{GRM}}$.

### **Accuracy of within-population genomic prediction with two separate**${\mathbf{GRM}}$**(WPMG)**

The within-population, multiple ${\mathbf{GRM}}$ model (WPMG) can be represented as:

2 $${\mathbf{y}}_{A} = 1\mu_{A} + {\mathbf{W}}_{{1_{A} }} {\mathbf{EGV}}_{{1_{A} }} + {\mathbf{W}}_{{2_{A} }} {\mathbf{EGV}}_{{2_{A} }} + {\mathbf{e}}_{A} .$$

With the WPMG model, two different sets of estimated genomic values are obtained for the validation candidates. These are ${\mathbf{EGV}}_{{A_{1} }}$, due to ${\mathbf{GRM}}_{1}$, and ${\mathbf{EGV}}_{{A_{2} }}$, due to ${\mathbf{GRM}}_{2}$. Both estimates of genomic values can be combined as sources of information in a selection index approach to obtain ${\mathbf{EGV}}_{{A_{T} }}$ as follows:

3 $${\mathbf{EGV}}_{{A_{T} }} = b_{{A_{1} }} {\mathbf{EGV}}_{{A_{1} }} + b_{{A_{2} }} {\mathbf{EGV}}_{{A_{2} }} ,$$

where $b_{{A_{1} }}$ and $b_{{A_{1} }}$ are weighting factors for the two sources of information. In the context of selection index theory, the breeding goal ($H$) is ${\mathbf{TGV}}_{A}$ and the index trait ($I$) is ${\mathbf{EGV}}_{{A_{T} }}$. The optimum values of $b_{{A_{1} }}$ and $b_{{A_{2} }}$ can be obtained as:

4 $$\left[ {\begin{array}{*{20}c} {b_{{A_{1} }} } \\ {b_{{A_{2} }} } \\ \end{array} } \right] = {\mathbf{P}}^{ - 1} {\mathbf{g}},$$

where ${\mathbf{P}}$ is the (co)variance matrix of information sources ${\mathbf{EGV}}_{{A_{1} }}$ and ${\mathbf{EGV}}_{{A_{2} }}$, and ${\mathbf{g}}$ is a vector containing the covariances between information sources ${\mathbf{EGV}}_{{A_{1} }}$ and ${\mathbf{EGV}}_{{A_{2} }}$ and the true genomic value (${\mathbf{TGV}}_{\text{A}}$). Thus:

5 $${\mathbf{P}} = \left[ {\begin{array}{ll} {var\left( {{\mathbf{EGV}}_{{A_{1} }} } \right)} & {cov\left( {{\mathbf{EGV}}_{{A_{1} }} ,{\mathbf{EGV}}_{{A_{2} }} } \right)} \\ {cov\left( {{\mathbf{EGV}}_{{A_{1} }} ,{\mathbf{EGV}}_{{A_{2} }} } \right)} & {var\left( {{\mathbf{EGV}}_{{A_{2} }} } \right)} \\ \end{array} } \right],$$

and

6 $${\mathbf{g}} = \left[ {\begin{array}{*{20}c} {cov\left( {{\mathbf{EGV}}_{{A_{1} }} ,{\mathbf{TGV}}_{\text{A}} } \right)} \\ {cov\left( {{\mathbf{EGV}}_{{A_{2} }} ,{\mathbf{TGV}}_{\text{A}} } \right)} \\ \end{array} } \right].$$

For simplicity of the derivation, we will assume that ${\mathbf{TGV}}_{\text{A}}$ are scaled such that they have a variance of 1. Therefore, the variance of the estimated genomic values can be calculated as the reliability ($r^{2}$) of the estimated genomic values.

7 $${\text{Thus}},\;\;var\left( {{\mathbf{EGV}}_{{A_{1} }} } \right) = r_{{{\mathbf{EGV}}_{{A_{1} }} }}^{2} \;\;{\text{and}}\;\;var\left( {{\mathbf{EGV}}_{{A_{2} }} } \right) = r_{{{\mathbf{EGV}}_{{A_{2} }} }}^{2} .$$

We assume that there is no covariance between ${\mathbf{EGV}}_{{A_{1} }}$ and ${\mathbf{EGV}}_{{A_{2} }}$, since the expectation is zero when both ${\mathbf{GRM}}$ are jointly fitted [25]. Thus, when ${\mathbf{GRM}}$ are fitted simultaneously in a GREML model, only partial variances are explained by each of the ${\mathbf{GRM}}$ such that the sum of the variances explained by the two ${\mathbf{GRM}}$ equals the total genetic variance for the trait that can be explained by all SNPs. The partial variances explained by SNPs in each ${\mathbf{GRM}}$ can be viewed as partial regression coefficients in a multiple regression scenario.

$${\text{Hence}},\;\;{\mathbf{P}} = \left[ {\begin{array}{*{20}c} {r_{{{\mathbf{EGV}}_{{A_{1} }} }}^{2} } & 0 \\ 0 & {r_{{{\mathbf{EGV}}_{{A_{2} }} }}^{2} } \\ \end{array} } \right].$$

The first element of the vector ${\mathbf{g}}$ is:

8 $$\begin{aligned} cov\left( {{\mathbf{EGV}}_{{A_{1} }} ,{\mathbf{TGV}}_{\text{A}} } \right) & = cor\left( {{\mathbf{EGV}}_{{A_{1} }} ,{\mathbf{TGV}}_{\text{A}} } \right)\sqrt {var\left( {{\mathbf{EGV}}_{{A_{1} }} } \right)*var\left( {{\mathbf{TGV}}_{\text{A}} } \right)} \hfill \\ & = \left( {r_{{{\mathbf{EGV}}_{{A_{1} }} }} } \right)\sqrt {r_{{{\mathbf{EGV}}_{{A_{1} }} }}^{2} *1} = r_{{{\mathbf{EGV}}_{{A_{1} }} }}^{2} \hfill \\ \end{aligned}$$

$${\text{Similarly}},\;\;cov\left( {{\mathbf{EGV}}_{{A_{2} }} ,{\mathbf{TGV}}_{\text{A}} } \right) = r_{{{\mathbf{EGV}}_{{A_{2} }} }}^{2} .$$

Therefore,

9 $${\mathbf{g}} = \left[ {\begin{array}{*{20}c} {r_{{{\mathbf{EGV}}_{{A_{1} }} }}^{2} } \\ {r_{{{\mathbf{EGV}}_{{A_{2} }} }}^{2} } \\ \end{array} } \right].$$

The accuracy of selection index, representing the accuracy of $${\mathbf{EGV}}_{{A_{T} }}$$ is the correlation between the index values and the breeding goal ($$r_{IH}$$). Thus, $$r_{IH} = \frac{{cov_{IH} }}{{\sigma_{I} \sigma_{H} }}$$. As explained in Falconer and MacKay [26], the selection index is constructed such that one unit of the index is equivalent to one unit of the breeding goal. In other words, the selection index is constructed such that the regression of the breeding goal on the index ($$b_{HI} )$$ is 1, resulting in the expression $$cov_{IH} = \sigma_{I}^{2}$$. Thus,

10 $$\begin{aligned} r_{IH} & = r_{{{\mathbf{EGV}}_{{A_{T} }} }} = \frac{{\sigma_{I} }}{{\sigma_{H} }} \hfill \\ & = \sqrt {\frac{{{\mathbf{b^{\prime}g}}}}{{var\left( {\text{H}} \right)}}} = \sqrt {\frac{{{\mathbf{g^{\prime}P}}^{ - 1} {\mathbf{g}}}}{{var\left( {{\mathbf{TGV}}_{A} } \right)}}} = \sqrt {{\mathbf{g^{\prime}P}}^{ - 1} {\mathbf{g}}} \hfill \\ & = \sqrt {\left[ {\begin{array}{*{20}c} {r_{{{\mathbf{EGV}}_{{A_{1} }} }}^{2} } & {r_{{{\mathbf{EGV}}_{{A_{2} }} }}^{2} } \\ \end{array} } \right]\left[ {\begin{array}{*{20}c} {r_{{{\mathbf{EGV}}_{{A_{1} }} }}^{2} } & 0 \\ 0 & {r_{{{\mathbf{EGV}}_{{A_{2} }} }}^{2} } \\ \end{array} } \right]^{ - 1} \left[ {\begin{array}{*{20}c} {r_{{{\mathbf{EGV}}_{{A_{1} }} }}^{2} } \\ {r_{{{\mathbf{EGV}}_{{A_{2} }} }}^{2} } \\ \end{array} } \right]} \hfill \\ & = \sqrt {\left[ {\begin{array}{*{20}c} {r_{{{\mathbf{EGV}}_{{A_{1} }} }}^{2} } & {r_{{{\mathbf{EGV}}_{{A_{2} }} }}^{2} } \\ \end{array} } \right]\left[ {\begin{array}{*{20}c} {\frac{{r_{{{\mathbf{EGV}}_{{A_{2} }} }}^{2} }}{{r_{{{\mathbf{EGV}}_{{A_{1} }} }}^{2} r_{{{\mathbf{EGV}}_{{A_{2} }} }}^{2} }}} & 0 \\ 0 & {\frac{{r_{{{\mathbf{EGV}}_{{A_{1} }} }}^{2} }}{{r_{{{\mathbf{EGV}}_{{A_{1} }} }}^{2} r_{{{\mathbf{EGV}}_{{A_{2} }} }}^{2} }}} \\ \end{array} } \right]\left[ {\begin{array}{*{20}c} {r_{{{\mathbf{EGV}}_{{A_{1} }} }}^{2} } \\ {r_{{{\mathbf{EGV}}_{{A_{2} }} }}^{2} } \\ \end{array} } \right]} \hfill \\ & = \sqrt {r_{{{\mathbf{EGV}}_{{A_{1} }} }}^{2} + r_{{{\mathbf{EGV}}_{{A_{2} }} }}^{2} } . \hfill \\ \end{aligned}$$

The accuracies of estimated genomic values $r_{{{\mathbf{EGV}}_{{A_{1} }} }}$ and $r_{{{\mathbf{EGV}}_{{A_{2} }} }}$ can be calculated as $\rho_{{A_{1} }} r_{{SNP_{{A_{1} }} }}$ and $\rho_{{A_{2} }} r_{{SNP_{{A_{2} }} }}$ respectively, where $r_{{SNP_{{A_{1} }} }}$ and $r_{{SNP_{{A_{2} }} }}$ are the accuracies of estimated SNP effects in population $A$ for SNPs in ${\mathbf{GRM}}_{1}$ and ${\mathbf{GRM}}_{2}$ respectively, $\rho_{{A_{1} }}$ and $\rho_{{A_{2} }}$ are the square root of the proportions of genetic variance explained in the validation population $A$ by SNPs in ${\mathbf{GRM}}_{1}$ and ${\mathbf{GRM}}_{2} ,$ respectively. The accuracies of estimated SNP effects $r_{{SNP_{{A_{1} }} }}$ and $r_{{SNP_{{A_{2} }} }}$ can be deterministically predicted by Daetwyler’s equation [6]. Hence, Eq. (10) can be written as:

11 $$\begin{aligned} r_{{{\mathbf{EGV}}_{{A_{T} }} }} & = \sqrt {\rho_{{A_{1} }}^{2} r_{{SNP_{{A_{1} }} }}^{2} + \rho_{{A_{2} }}^{2} r_{{SNP_{{A_{2} }} }}^{2} } \hfill \\ & = \sqrt {\rho_{{A_{1} }}^{2} \frac{{h_{A}^{2} N_{A} }}{{h_{A}^{2} N_{A} + M_{{e_{{A_{1} }} }} }} + \rho_{{A_{2} }}^{2} \frac{{h_{A}^{2} N_{A} }}{{h_{A}^{2} N_{A} + M_{{e_{{A_{2} }} }} }}} , \hfill \\ \end{aligned}$$

where $h_{A}^{2}$ is the heritability of the trait in population $A$, $N_{A}$ is the number of individuals from population $A$ in the training population, $M_{{e_{{_{{A_{1} }} }} }}$ and $M_{{e_{{A_{2} }} }}$ are the effective number of chromosome segments segregating in population $A$, based on variation in ${\mathbf{GRM}}_{1}$ and ${\mathbf{GRM}}_{2} ,$ respectively. The $M_{e}$ values represent the effective number of effects that are estimated in the model. The values for $M_{e}$ can be calculated as the inverse of the variance of within-population ${\mathbf{GRM}}$ [27, 28].

### Accuracy of multi-population GP with two separate ${\mathbf{GRM}}$ (MPMG model)

In the case of multi-population GP model with two ${\mathbf{GRM}}$ fitted simultaneously, we assume that individuals from two populations $A$ and $B$ are combined in a training population to estimate the total genomic values for validation candidates from population $A$ (${\mathbf{EGV}}_{{A_{T} }} )$. To estimate the accuracy of ${\mathbf{EGV}}_{{A_{T} }}$ from this model, we estimate the accuracy of a selection index in which ${\mathbf{EGV}}$ for the selection candidates are combined from two different models having either population $A$ or $B$ as training population. The first model is a WPMG model using individuals from population $A$ in the training population (${\mathbf{EGV}}_{{A_{1,A} }}$ and ${\mathbf{EGV}}_{{A_{2} ,A}}$). The second model is an across-population model with two ${\mathbf{GRM}}$ using individuals from population $B$ in the training population (${\mathbf{EGV}}_{{A_{1,B} }}$ and ${\mathbf{EGV}}_{{A_{2} ,B}}$). The selection index was as follows:

12 $$\begin{aligned}{\mathbf{EGV}}_{{A_{T} }} & = b_{{A_{1} ,A}} {\mathbf{EGV}}_{{A_{1} ,A}} + b_{{A_{1} ,B}} {\mathbf{EGV}}_{{A_{1} ,B}} \\ & \quad + b_{{A_{2} ,A}} {\mathbf{EGV}}_{{A_{2} ,A}} + b_{{A_{2} ,B}} {\mathbf{EGV}}_{{A_{2} ,B}} . \end{aligned}$$

The (co)variance matrix of information sources is given as:

13 $${\mathbf{P}} = \left[ {\begin{array}{ll} {var\left( {{\mathbf{EGV}}_{{A_{1} ,A}} } \right)} \\ {cov\left( {{\mathbf{EGV}}_{{A_{1} ,A}} ,{\mathbf{EGV}}_{{A_{1} ,B}} } \right)} \\ 0 \\ 0 \\ \end{array} \begin{array}{ll} { cov\left( {{\mathbf{EGV}}_{{A_{1} ,A}} ,{\mathbf{EGV}}_{{A_{1} ,B}} } \right)} \\ {var\left( {{\mathbf{EGV}}_{{A_{1} ,B}} } \right)} \\ 0 \\ 0 \\ \end{array} \begin{array}{ll} 0 \\ 0 \\ {var\left( {{\mathbf{EGV}}_{{A_{2} ,A}} } \right)} \\ {cov\left( {{\mathbf{EGV}}_{{A_{2} ,A}} ,{\mathbf{EGV}}_{{A_{2} ,B}} } \right)} \\ \end{array} \begin{array}{ll} 0 \\ 0 \\ {cov\left( {{\mathbf{EGV}}_{{A_{2} ,A}} ,{\mathbf{EGV}}_{{A_{2} ,B}} } \right)} \\ {var\left( {{\mathbf{EGV}}_{{A_{2} ,B}} } \right)} \\ \end{array} } \right].$$

Again, we assume that the covariances between ${\mathbf{EGV}}$ based on ${\mathbf{GRM}}_{1}$ and ${\mathbf{GRM}}_{2}$ are zero. Given also that we assume a variance of 1 for ${\mathbf{TGV}}_{A}$:

$$var\left( {{\mathbf{EGV}}_{{A_{1} ,A}} } \right) = r_{{{\mathbf{EGV}}_{{A_{1} ,A}} }}^{2} ,$$

$$var\left( {{\mathbf{EGV}}_{{A_{1} ,B}} } \right) = r_{{{\mathbf{EGV}}_{{A_{1} ,B}} }}^{2} ,$$

$$var\left( {{\mathbf{EGV}}_{{A_{2} ,A}} } \right) = r_{{{\mathbf{EGV}}_{{A_{2} ,A}} }}^{2} ,$$

$$var\left( {{\mathbf{EGV}}_{{A_{2} ,B}} } \right) = r_{{{\mathbf{EGV}}_{{A_{2} ,B}} }}^{2} .$$

$cov\left( {{\mathbf{EGV}}_{{A_{1} ,A}} ,{\mathbf{EGV}}_{{A_{1} ,B}} } \right)$ is the covariance of the ${\mathbf{EGV}}_{{A_{1} }}$ calculated using SNP effects estimated in the reference populations $A$ and $B$, respectively. Following the derivation in Appendix 1, we show that:

$$cov\left( {{\mathbf{EGV}}_{{A_{1} ,A}} ,{\mathbf{EGV}}_{{A_{1} ,B}} } \right) = r_{{{\mathbf{EGV}}_{{A_{1} ,A}} }}^{2} r_{{{\mathbf{EGV}}_{{A_{1} ,B}} }}^{2} ,$$

$${\text{and}}\;cov\left( {{\mathbf{EGV}}_{{A_{2} ,A}} ,{\mathbf{EGV}}_{{A_{2} ,B}} } \right) = r_{{{\mathbf{EGV}}_{{A_{2} ,A}} }}^{2} r_{{{\mathbf{EGV}}_{{A_{2} ,B}} }}^{2} .$$

Therefore,

14 $${\mathbf{P}} = \left[ {\begin{array}{*{20}c} {r_{{{\mathbf{EGV}}_{{A_{1} ,A}} }}^{2} } \\ {r_{{{\mathbf{EGV}}_{{A_{1} ,A}} }}^{2} r_{{{\mathbf{EGV}}_{{A_{1} ,B}} }}^{2} } \\ 0 \\ 0 \\ \end{array} \begin{array}{*{20}c} {r_{{{\mathbf{EGV}}_{{A_{1} ,A}} }}^{2} r_{{{\mathbf{EGV}}_{{A_{1} ,B}} }}^{2} } \\ {r_{{{\mathbf{EGV}}_{{A_{1} ,B}} }}^{2} } \\ 0 \\ 0 \\ \end{array} \begin{array}{*{20}c} 0 \\ 0 \\ {r_{{{\mathbf{EGV}}_{{A_{2} ,A}} }}^{2} } \\ {r_{{{\mathbf{EGV}}_{{A_{2} ,A}} }}^{2} r_{{{\mathbf{EGV}}_{{A_{2} ,B}} }}^{2} } \\ \end{array} \begin{array}{*{20}c} 0 \\ 0 \\ {r_{{{\mathbf{EGV}}_{{A_{2} ,A}} }}^{2} r_{{{\mathbf{EGV}}_{{A_{2} ,B}} }}^{2} } \\ {r_{{{\mathbf{EGV}}_{{A_{2} ,B}} }}^{2} } \\ \end{array} } \right] .$$

Following Eq. (9), ${\mathbf{g}}$ can be written as:

15 $${\mathbf{g}} = \left[ {\begin{array}{*{20}c} {\begin{array}{*{20}c} {r_{{{\mathbf{EGV}}_{{A_{1} ,A}} }}^{2} } \\ {r_{{{\mathbf{EGV}}_{{A_{1} ,B}} }}^{2} } \\ \end{array} } \\ {\begin{array}{*{20}c} {r_{{{\mathbf{EGV}}_{{A_{2} ,A}} }}^{2} } \\ {r_{{{\mathbf{EGV}}_{{A_{2} ,B}} }}^{2} } \\ \end{array} } \\ \end{array} } \right].$$

The accuracy of the index, representing $r_{{{\hat{\text{g}}}_{{A_{T} }} }}$ can be calculated as:

$$r_{{{\mathbf{EGV}}_{{A_{T} }} }} = \sqrt {\frac{{{\mathbf{g^{\prime}P}}^{ - 1} {\mathbf{g}}}}{{var\left( {{\mathbf{TGV}}_{A} } \right)}}} = \sqrt {{\mathbf{g^{\prime}P}}^{ - 1} {\mathbf{g}}} .$$

With some algebra (see Appendix 2), we show that the equation $\sqrt {{\mathbf{g^{\prime}P}}^{ - 1} {\mathbf{g}}}$ for the MPMG model can be represented as:

16 $$\sqrt{\begin{array}{c}{\rho }_{{A}_{1}}^{2}\left(\frac{\frac{{h}_{A}^{2}}{{ME}_{{A}_{1}}}\left(\frac{{h}_{B}^{2}}{{{M}_{e}}_{{AB}_{1}}}+\frac{1}{{N}_{B}}\right)+{r}_{g}^{2}\frac{{h}_{B}^{2}}{{{M}_{e}}_{{AB}_{1}}}\left(\frac{{h}_{A}^{2}}{{{M}_{e}}_{{A}_{1}}}+\frac{1}{{N}_{A}}\right)-2\left(\frac{{h}_{A}^{2}}{{{M}_{e}}_{{A}_{1}}}\right)\left({r}_{g}^{2}\frac{{h}_{B}^{2}}{{{M}_{e}}_{{AB}_{1}}}\right)}{\left(\frac{{h}_{A}^{2}}{{{M}_{e}}_{{A}_{1}}}+\frac{1}{{N}_{A}}\right)\left(\frac{{h}_{B}^{2}}{{{M}_{e}}_{{AB}_{1}}}+\frac{1}{{N}_{B}}\right)-\left(\frac{{h}_{A}^{2}}{{{M}_{e}}_{{A}_{1}}}\right)\left({r}_{g}^{2}\frac{{h}_{B}^{2}}{{{M}_{e}}_{{AB}_{1}}}\right)}\right)\\ + {\rho }_{{A}_{2}}^{2}\left(\frac{\frac{{h}_{A}^{2}}{{{M}_{e}}_{{A}_{2}}}\left(\frac{{h}_{B}^{2}}{{{M}_{e}}_{{AB}_{2}}}+\frac{1}{{N}_{B}}\right)+{r}_{g}^{2}\frac{{h}_{B}^{2}}{{{M}_{e}}_{{AB}_{2}}}\left(\frac{{h}_{A}^{2}}{{{M}_{e}}_{{A}_{2}}}+\frac{1}{{N}_{A}}\right)-2\left(\frac{{h}_{A}^{2}}{{{M}_{e}}_{{A}_{2}}}\right)\left({r}_{g}^{2}\frac{{h}_{B}^{2}}{{{M}_{e}}_{{AB}_{2}}}\right)}{\left(\frac{{h}_{A}^{2}}{{{M}_{e}}_{{A}_{2}}}+\frac{1}{{N}_{A}}\right)\left(\frac{{h}_{B}^{2}}{{{M}_{e}}_{{AB}_{2}}}+\frac{1}{{N}_{B}}\right)-\left(\frac{{h}_{A}^{2}}{{{M}_{e}}_{{A}_{2}}}\right)\left({r}_{g}^{2}\frac{{h}_{B}^{2}}{{{M}_{e}}_{{AB}_{2}}}\right)}\right)\end{array}},$$

which in matrix form can be represented as:

17 $$\sqrt{{\left[\begin{array}{c}\begin{array}{c}{\rho }_{{A}_{1}}\sqrt{\frac{{h}_{A}^{2}}{{{M}_{e}}_{{A}_{1}}}}\\ {\rho }_{{A}_{1}}{r}_{g}\sqrt{\frac{{h}_{B}^{2}}{{{M}_{e}}_{{AB}_{1}}}}\end{array}\\ \begin{array}{c}{\rho }_{{A}_{2}}\sqrt{\frac{{h}_{A}^{2}}{{{M}_{e}}_{{A}_{2}}}}\\ {\rho }_{{A}_{2}}{r}_{g}\sqrt{\frac{{h}_{B}^{2}}{{{M}_{e}}_{{AB}_{2}}}}\end{array}\end{array}\right]}^{T}{\left[\begin{array}{c}\frac{{{h}^{2}}_{A}}{{{M}_{e}}_{{A}_{1}}}+\frac{1}{{N}_{A}}\\ {r}_{g}\frac{\sqrt{{h}_{A}^{2}{h}_{B}^{2}}}{\sqrt{{{M}_{e}}_{{A}_{1}}{{M}_{e}}_{{AB}_{1}}}}\\ 0\\ 0\end{array}\begin{array}{c}{r}_{g}\frac{\sqrt{{h}_{A}^{2}{h}_{B}^{2}}}{\sqrt{{{M}_{e}}_{{A}_{1}}{{M}_{e}}_{{AB}_{1}}}}\\ \frac{{{h}^{2}}_{B}}{{{M}_{e}}_{{AB}_{1}}}+\frac{1}{{N}_{B}}\\ 0\\ 0\end{array}\begin{array}{c}0\\ 0\\ \frac{{{h}^{2}}_{A}}{{{M}_{e}}_{{A}_{2}}}+\frac{1}{{N}_{A}}\\ {r}_{g}\frac{\sqrt{{h}_{A}^{2}{h}_{B}^{2}}}{\sqrt{{{M}_{e}}_{{A}_{2}}{{M}_{e}}_{{AB}_{2}}}}\end{array}\begin{array}{c}0\\ 0\\ {r}_{g}\frac{\sqrt{{h}_{A}^{2}{h}_{B}^{2}}}{\sqrt{{{M}_{e}}_{{A}_{2}}{{M}_{e}}_{{AB}_{2}}}}\\ \frac{{h}_{B}^{2}}{{{M}_{e}}_{{AB}_{2}}}+\frac{1}{{N}_{B}}\end{array}\right]}^{-1}\left[\begin{array}{c}\begin{array}{c}{\rho }_{{A}_{1}}\sqrt{\frac{{h}_{A}^{2}}{{{M}_{e}}_{{A}_{1}}}}\\ {\rho }_{{A}_{1}}{r}_{g}\sqrt{\frac{{h}_{B}^{2}}{{{M}_{e}}_{{AB}_{1}}}}\end{array}\\ \begin{array}{c}{\rho }_{{A}_{2}}\sqrt{\frac{{h}_{A}^{2}}{{{M}_{e}}_{{A}_{2}}}}\\ {\rho }_{{A}_{2}}{r}_{g}\sqrt{\frac{{h}_{B}^{2}}{{{M}_{e}}_{{AB}_{2}}}}\end{array}\end{array}\right]}$$

The input parameters are:

$h_{A}^{2}$ =  heritability of the trait in population $A$, $h_{B}^{2}$ =  heritability of the trait in population $B$, $N_{A}$ = number of individuals from population $A$ in the training population, $N_{B}$ = number of individuals from population $B$ in the training population, $\rho_{{A_{1} }}$ =  square root of the proportion of genetic variance explained in the validation population $A$ by ${\mathbf{GRM}}_{1}$, $\rho_{{A_{2} }}$ =  square root of the proportion of genetic variance explained in the validation population $A$ by ${\mathbf{GRM}}_{2}$, $r_{g}$  = genetic correlation between populations $A$ and $B$, $M_{{e_{{A_{1} }} }}$  = number of effective chromosome segments in population $A$ based on variation in ${\mathbf{GRM}}_{1}$, $M_{{e_{{A_{2} }} }}$ =  number of effective chromosome segments in population $A$ based on variation in ${\mathbf{GRM}}_{2}$, $M_{{e_{{AB_{1} }} }}$  = number of effective chromosome segments across populations $A$ and $B$ based on variation in ${\mathbf{GRM}}_{1}$, $M_{{e_{{AB_{2} }} }}$  =  number of effective chromosome segments across populations $A$ and $B$ based on variation in ${\mathbf{GRM}}_{2}$.

For predicting $r_{{{\mathbf{EGV}}_{{A_{T} }} }}$, Eq. (17) reduces to Eq. (11) for within-population GP when $r_{g}$ between populations $A$ and $B$ is 0. The values for $M_{{e_{AB} }}$ can be calculated as the inverse of the variance of the across-population block of multi-population ${\mathbf{GRM}}$ [16, 27].

### Validation of prediction equations using simulations

The aim of this section was to use simulations to validate Eqs. 11 (WPMG) and 17 (MPMG) in scenarios that differed in the proportion of causal SNPs that are pre-selected and fitted in the models. Consequently, the scenarios differed in the proportion of total genetic variance explained by SNPs in each of the two ${\mathbf{GRM}}$ fitted simultaneously in the models. The scenarios also differed in heritability of the trait. For the MPMG model, the scenarios also differed in the level of genetic correlation between the populations $A$ and $B$. Genotype data of two existing cattle populations were used in combination with simulated phenotypes. As validation, we compared the empirical accuracies in each simulated scenario to the accuracy obtained using the derived prediction equations.

### Genotype data

Genotypes for SNPs on the Illumina Bovinesnp50 (Illumina Inc., San Diego, CA, USA) with 48,912 SNPs after quality control, were available on 595 New Zealand Jersey bulls and 5553 Dutch Holstein bulls. These SNPs had at least ten copies of the minor allele in a combined Dutch Holstein and New Zealand Jersey population, with a minor allele frequency (MAF) ranging from 0.009 to 0.5. Hereafter, we will refer to the New Zealand Jerseys as population $A$ and to the Dutch Holsteins as population $B$.

### Simulation of phenotypes

Phenotypes for all individuals were simulated using their real genotypes and assuming an additive model. From the 48,912 SNPs, 500 were randomly selected to be causal SNPs in both populations. Allele substitution effect of the causal SNPs ($$a)$$ were sampled from a bi-variate normal distribution with a mean of 0, variance of 1, and a correlation of 0.8, 0.6 and 0.4 between populations $$A$$ and $$B$$. Since allele substitution effects were sampled independently from their allele frequency, the correlation between allele substitution effect represents the correlation between genomic values of individuals from populations $$A$$ and $$B$$, which is referred to as the genetic correlation between populations ($$r_{g}$$). Within each population, $${\mathbf{TGV}}$$ for individual $$i$$ were calculated as $$\sum \left( {x_{i,j} *a_{j} } \right)$$, where $$x_{i,j}$$ is the genotype of individual $$i$$ at causal SNP $$j$$ (coded as 0, 1, 2) and $$a_{j}$$ is the allele substitution effect of causal SNP $$j$$. The corresponding phenotype was computed as $${\mathbf{TGV}}_{i} + e_{i}$$, where $$e_{i}$$ is the residual effect of individual $$i$$, sampled from a standard normal distribution with a mean of 0 and a variance equal to $$\sigma_{{g_{k} }}^{2} *\left( {\frac{1}{{h^{2} }} - 1} \right)$$, where $$\sigma_{{g_{k} }}^{2}$$ is the variance of $${\mathbf{TGV}}$$ in population $$k$$. For each population, the residual effects were sampled from a separate normal distribution. The heritability ($$h^{2}$$) was set to 0.3 and 0.8 in each population. Simulation of phenotypes was carried out in R [29] and was replicated 100 times.

### Genomic prediction

The WPMG (only 476 individuals from population $A$ in the training population) and MPMG (476 individuals from population $A$ and 5553 individuals from population $B$ in the training population) models were implemented in the software MTG2 [30]. We used three levels for the proportion of causal SNPs that are identified, pre-selected and used to create the first ${\mathbf{GRM}}$. The number of causal SNPs underlying the simulated trait was always 500. The levels are:

- CSNP_125: this level represents a situation in which a quarter of the causal SNPs are identified, pre-selected and used to create the first ${\mathbf{GRM}}$.
- CSNP_250: this level represents a situation in which half of the causal SNPs are identified, pre-selected and used to create the first ${\mathbf{GRM}}$.
- CSNP_500: this level represents the extreme situation in which all 500 causal SNPs are identified, pre-selected and used to create the first ${\mathbf{GRM}}$.

At all levels, the remaining SNPs that were not used as causal were used to create the second ${\mathbf{GRM}}$. The level above were evaluated under varying degrees of genetic correlation between populations (0.4, 0.6 and 0.8) and heritability in both populations (0.3 and 0.8). Throughout the study, individuals from population $A$ were used as the validation candidates in a fivefold cross-validation scheme, where individuals from population $A$ were randomly split into five sets of 119 individuals each. The ${\mathbf{GRM}}$ fitted in the MPMG model were constructed according to Wientjes et al. [21], considering population-specific allele frequencies. The empirical accuracies of prediction at all levels were computed as the correlation between the ${\mathbf{EGV}}$ and the simulated ${\mathbf{TGV}}$ of validation candidates.

To compare how accurate the accuracy of the MPMG model can be predicted compared to a MPSG model, we also fitted a multi-population, single ${\mathbf{GRM}}$ (MPSG) model, with the multi-population ${\mathbf{GRM}}$ made from non-causal SNPs in addition to either the CSNP_125, CSNP_250 or CSNP_500 SNPs. To predict the accuracy of the MPSG model, we used the derived prediction Eq. (17) in which $\rho_{{A_{2} }}^{2}$ is set to 0, given that the model has only one ${\mathbf{GRM}}$. The value used for $\rho_{{A_{1} }}^{2}$ was empirically estimated as the ratio of empirical accuracy of the WPSG model and the predicted accuracy using the formula of Daetwyler et al. [6], assuming all the variance is captured by SNPs. Setting $\rho_{{A_{2} }}^{2}$ to 0 reduces Eq. (17) to the equation derived by Wientjes et al. [16].

### Values of input parameters for the prediction equations

For the prediction of accuracy using Eqs. (11) and (17), we used the simulated values as input for the parameters $h_{A}^{2}$, $h_{B}^{2}$, and $r_{g}$. The values used for $\rho_{{A_{1} }}^{2}$ were 0.25, 0.5 and 1 in the CSNP_125, CSNP_250 and CSNP_500 levels, respectively. This is because a quarter of the causal SNPs explains, on average, a quarter of the total genetic variance for the trait, which was confirmed empirically. An empirical approach was used to determine the appropriate values for the input parameter $\rho_{{A_{2} }}^{2}$. This parameter represents the proportion of total genetic variance of the trait in the validation population explained by the non-causal SNPs (${\mathbf{GRM}}_{2}$) in the training population. We determined empirically that the non-causal SNPs could only explain 66% of the total genetic variance of the trait in the validation individuals of population $A$ using a within-population model with one ${\mathbf{GRM}}$ including all non-causal SNPs. We did this by taking a ratio of the empirical accuracy obtained from cross-validation and the predicted accuracy using Daetwyler’s equation [6], assuming that 100% of the total genetic variance for the trait is captured by SNPs. Thus, the values for $\rho_{{A_{2} }}^{2}$ as used in the prediction equation were 0.66 × 0.75, 0.66x0.5 and 0, in the CSNP_125, CSNP_250 and CSNP_500 levels, respectively, with the values 0.75, 0.5 and 0 representing the proportion of total genetic variance unexplained by the causal SNPs (${\mathbf{GRM}}_{1}$) in the CSNP_125, CSNP_250 and CSNP_500 levels, respectively. Throughout the study, $M_{e}$ within a population was calculated according to Lee et al. [28] as the inverse of the variance of the within-population ${\mathbf{GRM}}$, while $M_{e}$ across populations was calculated as the inverse of the variance of the across-population block terms of the multi-population ${\mathbf{GRM}}$ [16, 27].

### Potential accuracies of different models in relation to different levels of $\varvec{r}_{\varvec{g}}$, $\varvec{ \rho }_{{\varvec{A}_{1} }}$, and $\varvec{ME}_{{\varvec{AB}_{2} }}$

The aim of this section was to identify the situations under which the MPMG model will outperform all other models tested in terms of prediction accuracy. We evaluated the potential accuracy of predicting the genomic values of selection candidates from a numerically small population $A$ under three cases. These are hypothetical cases that aim to mimic real life situations in dairy cattle breeding programs.

#### Case 1

For the first case, we assume that, in addition to individuals from the target population $A$ (${\text{N}}_{\text{A}} = 476)$, individuals from a larger but different population $B$ (${\text{N}}_{\text{B}} = 5553$) are available to be included in the training population, mimicking the real sample sizes of the Jersey and Holstein populations used in this study. We investigated the effect of the level of genetic correlation between populations on the accuracy of prediction. The following additional assumptions were made: $M_{e}$ within population $A$ based on pre-selected SNPs (calculated from real genotype data) = 159; $M_{e}$ across populations $A$ and $B$ based on pre-selected SNPs (calculated from real genotype data) = 280; $M_{e}$ within population ${\text{A}}$ based on remaining SNPs (calculated from real genotype data) = 463; $M_{e}$ across populations $A$ and $B$ based on remaining SNPs (calculated from real genotype data) = 32,970; heritability of the trait: 0.3 in both populations; ${\rho }^{2}$ due to 500 pre-selected causal SNPs = 0.4, ${\rho }^{2}$ due to all SNPs = 0.8 (assuming the remaining non-causal SNPs capture 66% of the residual genetic variance).

#### Case 2

Many genome-wide association studies have been carried out in livestock with the aim to identify the causal variants underlying complex traits. The variants that were discovered explain varying proportions of the genetic variance for the traits of interest. Here, we evaluated the potential accuracy of prediction under situations ranging from poor causal SNP discovery (discovered “causal SNPs” explain 0% of genetic variance) to accurate causal SNP discovery (discovered causal SNPs explain 100% of genetic variance for the trait). The following additional assumptions were made: $M_{e}$ within population $A$ based on pre-selected SNPs (calculated from real genotype data) = 159; $M_{e}$ across populations $A$ and $B$ based on 500 pre-selected SNPs (calculated from real genotype data) = 280; $M_{e}$ within population $A$ based on 48,412 remaining SNPs (calculated from real genotype data) = 463; $M_{e}$ across populations $A$ and $B$ based on 48,412 remaining SNPs (calculated from real genotype data) = 32,970; genetic correlations between populations $A$ and $B$ = 0.6; heritability of the trait = 0.3 in both populations; proportion of genetic variance explained by all SNPs = 1.

#### Case 3

In this case, our aim was to explore the impact of the closeness between the validation population and the training populations as measured by the $M_{e}$ across populations. In most studies, the number of variants identified as causal for complex traits is at most a few hundred. In the context of the MPMG model, the $M_{e}$ across populations based on the identified potential causal SNPs is usually small, with an upper bound equal to the total number of identified “causal SNPs”. A parameter that is expected to be considerably large, especially with increasing SNP density is the $M_{e}$ based on the remaining unselected SNPs. Here, we evaluated the effect of $M_{e}$ across populations based on the remaining unselected SNPs on the potential accuracy of prediction. We varied $M_{e}$ across populations from 1000 to 50,000. The following additional assumptions were made: $M_{e}$ within population $A$ based on 500 pre-selected SNPs (calculated from real genotype data) = 159; $M_{e}$ across populations $A$ and $B$ based on 500 pre-selected SNPs (calculated from real genotype data) = 280; $M_{e}$ within population $A$ based on 48,412 remaining SNPs (calculated from real genotype data) = 463; genetic correlations between populations $A$ and $B$ = 0.6; heritability of the trait = 0.3 in both populations; $\rho^{2}$ due to 500 pre-selected causal SNPs = 0.4, $\rho^{2}$ due to all SNPs = 0.8 (assuming the remaining non-causal SNPs capture 66% of the residual genetic variance).

#### Case 4

The study of Van den Berg et al. [31], reported that $M_{e}$ values estimated from the ${\mathbf{GRM}}$ are underestimated by ~ 80% compared to $M_{e}$ values back-solved from the empirical accuracy of a GBLUP model. In case 4, we investigated the potential impact of an underestimation of $M_{e}$ within the predicted population $A$ on the accuracy of prediction. Thus, we used underestimated values of $M_{e}$ for both ${\mathbf{GRM}}_{1}$ and ${\mathbf{GRM}}_{2}$ in the prediction equations, with the extent of underestimation ranging from 0 to 90%. The following additional assumptions were made: $M_{e}$ across populations $A$ and $B$ based on 500 pre-selected SNPs (calculated from real genotype data) = 280; $M_{e}$ across populations $A$ and $B$ based on 48,412 remaining SNPs (calculated from real genotype data) = 32,970; genetic correlations between populations $A$ and $B$: = 0.6; heritability of the trait = 0.3 in both populations; proportion of genetic variance explained by SNPs in ${\mathbf{GRM}}_{1}$ = 0.5.

In all four cases, we evaluated the potential accuracy of three models using their prediction equations as follows.

*Within*-*population, single*-*GRM (WPSG) model*: To predict the potential accuracy of this model, we used the formula of Daetwyler et al. [6], which takes the proportion of genetic variance explained by all SNPs ($\rho_{A}^{2}$) into account, as $\sqrt {\rho_{A}^{2} \frac{{h_{A}^{2} N_{A} }}{{h_{A}^{2} N_{A} + M_{{e_{{A_{1} }} }} }}}$. The value for $M_{{e_{{A_{1} }} }}$ was calculated based on all SNPs.

*Within population, multiple GRM (WPMG) model*: to predict the potential accuracy of this model, we used the derived prediction Eq. (12).

*Multi*-*population, single GRM (MPSG) model*: to predict the potential accuracy of this model, we used the formula of Wientjes et al. [16]:

$$\sqrt{{\left[\begin{array}{c}{\rho }_{A}\sqrt{\frac{{h}_{A}^{2}}{{{M}_{e}}_{A}}}\\ {\rho }_{A}{r}_{g}\sqrt{\frac{{h}_{B}^{2}}{{{M}_{e}}_{AB}}}\end{array}\right]}^{T} {\left[\begin{array}{cc}\frac{{{h}^{2}}_{A}}{{{M}_{e}}_{{A}_{1}}}+\frac{1}{{N}_{A}}& {r}_{g}\frac{\sqrt{{h}_{A}^{2}{h}_{B}^{2}}}{\sqrt{{{M}_{e}}_{A}{{M}_{e}}_{AB}}}\\ {r}_{g}\frac{\sqrt{{h}_{A}^{2}{h}_{B}^{2}}}{\sqrt{{{M}_{e}}_{A}{{M}_{e}}_{AB}}}& \frac{{{h}^{2}}_{B}}{{{M}_{e}}_{AB}}+\frac{1}{{N}_{B}}\end{array}\right]}^{-1}\left[\begin{array}{c}{\rho }_{A}\sqrt{\frac{{h}_{A}^{2}}{{{M}_{e}}_{A}}}\\ {\rho }_{A}{r}_{g}\sqrt{\frac{{h}_{B}^{2}}{{{M}_{e}}_{AB}}}\end{array}\right]}$$

Here also, $M_{{e_{A} }}$ and $M_{{e_{AB} }}$ were calculated based on all SNPs.

*Multi*-*population, multiple GRM (MPMG) model*: to predict potential accuracy, we used the derived prediction Eq. (17).

## Results

### Number of independent chromosome segments ($\varvec{M}_{\varvec{e}}$) within and across populations

The number of independent chromosome segments per SNP set estimated within population $A$ (595 New Zealand Jersey) and across populations $A$ and $B$ (5553 Dutch Holsteins) are in Table 1.

**Table 1:** Number of independent chromosome segments (${\mathbf{M}}_{{\mathbf{e}}}$) per SNP set estimated within the target population $\varvec{A}$ and across populations $\varvec{A}$ and $\varvec{B}$

SNP set (number of SNPs)	$\varvec{M}_{\varvec{e}}$ within population $\varvec{A}$	$\varvec{M}_{\varvec{e}}$ across populations $\varvec{A}$ and $\varvec{B}$
Non-causal SNPs (48,412)	280	32,970
CSNPs_500 + non-causal SNPs (48,912)^a^	280	33,242
CSNPs_250 + non-causal SNPs (48,662)^a^	280	33,056
CSNPs_125 + non-causal SNPs (48,537)^a^	280	32,984
CSNPs_500 (500)	159	463
CSNPs_250 (250)	107	236
CSNPs_125 (125)	67	124

^a^Non-causal SNPs combined with the pre-selected SNPs in a single ${\mathbf{GRM}}$

The same value of $M_{e}$ within population $A$ (280) was obtained when all 48,912 SNPs and when only the non-causal SNPs were used to construct the ${\mathbf{GRM}}$. Estimated $M_{e}$ within population $A$ differed markedly between SNP sets only when the number of SNPs used to calculate the ${\mathbf{GRM}}$ was small. At lower SNP densities, $M_{e}$ across population $A$ and $B$ were close to the number of SNPs used to calculate the ${\mathbf{GRM}}$. $M_{e}$ across populations obtained with higher density SNPs were similar.

### Empirical versus predicted accuracies of the within-population, multiple ${\mathbf{GRM}}$ (WPMG) model

**Fig. 1:**
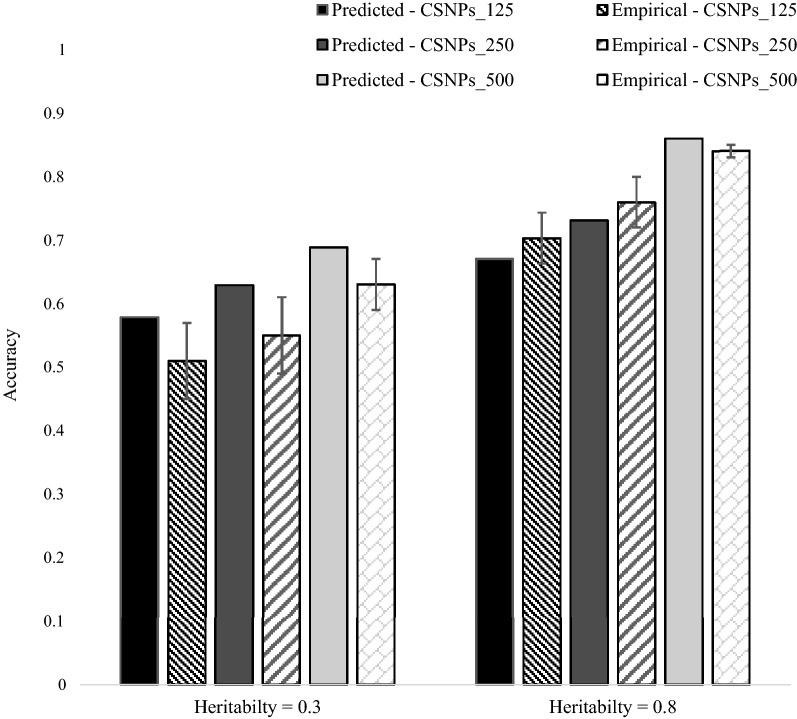
Predicted versus empirical accuracies of the within-population, multiple ${\mathbf{GRM}}$ genomic prediction model (476 individuals from population $A$ in the training population), with a simulated trait heritability of 0.3 and 0.8. The standard error bars represent twice the standard deviation of the accuracies across 100 replicates

Empirical and predicted accuracies of the WPMG model (476 individuals in the training population) under the different levels of pre-selection of causal SNPs, with a simulated trait heritability of 0.3 and 0.8 are in Fig. 1. For a low heritability trait (0.3), Eq. (11) over-predicts the empirical accuracy of the WPMG model, with the extent of over-prediction ranging from 8.4% (CSNP_500) to 11.8% (CSNP_125). For a high heritability trait (0.8), predicted accuracies were close to empirical accuracies, with the predicted accuracies within the standard errors of empirical accuracies (except for CSNP_500).

### Empirical versus predicted accuracies of the multi-population, single ${\mathbf{GRM}}$ (MPSG) model

**Fig. 2:**
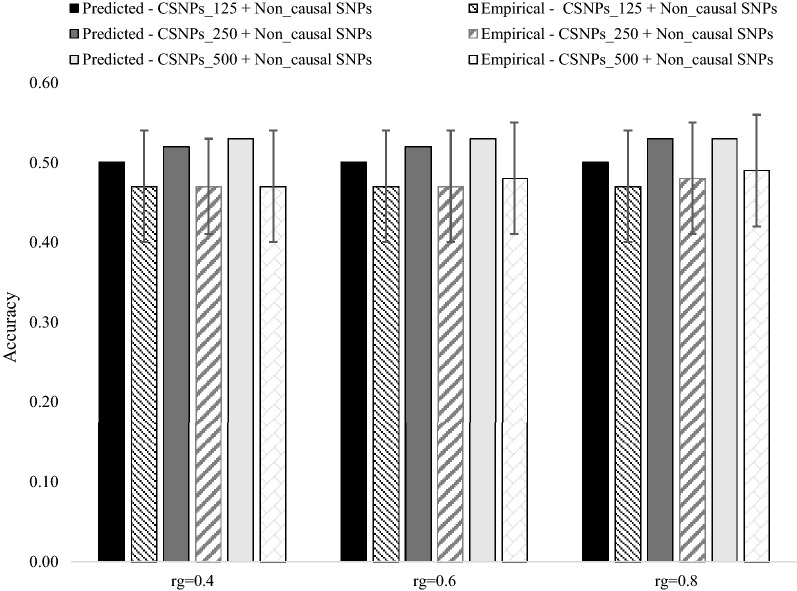
Predicted versus empirical accuracies of the multi-population, single ${\mathbf{GRM}}$ genomic prediction model (5553 individuals from population $\varvec{B}$ and 476 individuals from population $\varvec{A}$ in the training population) under the three different levels of inclusion of causal SNPs in the ${\mathbf{GRM}}$, with genetic correlation ($\varvec{r}_{\varvec{g}}$) between populations $\varvec{A}$ and $\varvec{B}$ of 0.4, 0.6 and 0.8, and a heritability of 0.3 in both populations. The standard error bars represent twice the standard deviation of the accuracies across 100 replicates

**Fig. 3:**
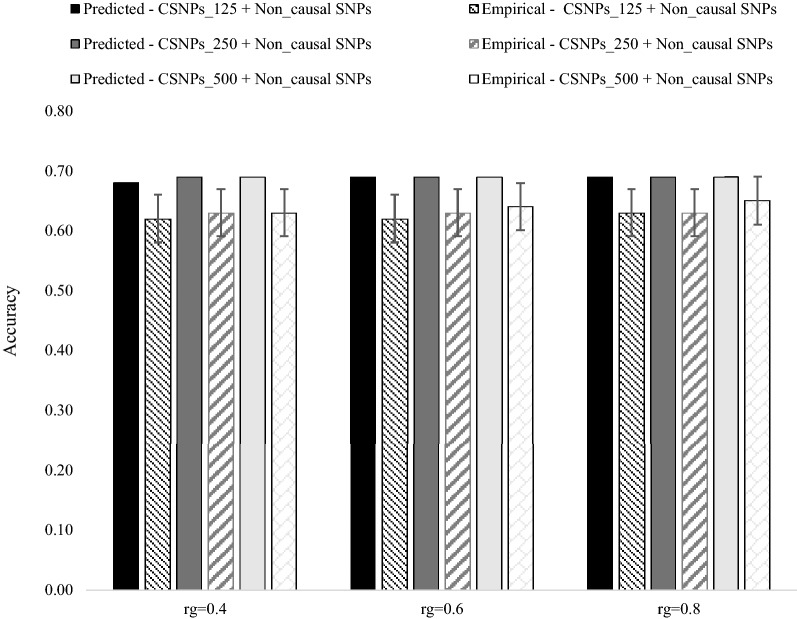
Predicted versus empirical accuracies of the multi-population, single ${\mathbf{GRM}}$ genomic prediction model (5553 individuals from population $\varvec{B}$ and 476 individuals from population $\varvec{A}$ in the training population) under the three different levels of inclusion of causal SNPs in the ${\mathbf{GRM}}$, with genetic correlation ($\varvec{r}_{\varvec{g}}$) between populations $\varvec{A}$ and $\varvec{B}$ of 0.4, 0.6 and 0.8, and a heritability of 0.8 in both populations. The standard error bars represent twice the standard deviation of the accuracies across 100 replicates

Empirical and predicted accuracies of the MPSG model under different levels of inclusion of causal SNPs in the ${\mathbf{GRM}}$ and different levels of $r_{g}$ between populations $A$ and *B* are shown in Fig. 2 (heritability in both populations = 0.3) and Fig. 3 (heritability in both populations = 0.8). As expected, given the large number of $M_{e}$ across populations, empirical accuracies were not significantly different between scenarios differing in $r_{g}$ and percentage of causal SNPs included in the ${\mathbf{GRM}}$. The standard errors of empirical accuracies were higher at low heritability (Fig. 2), than at high heritability (Fig. 3), which most likely reflects the higher level of noise in the phenotype at low heritability than at a high heritability. As expected, an increase in heritability resulted in an increase in empirical accuracies. In general, the prediction equation for the accuracy of the MPSG model resulted in an over-prediction of empirical accuracy ranging from ~ 5 to 10%.

### Empirical versus predicted accuracies of the multi-population, multiple ${\mathbf{GRM}}$ (MPMG) model

**Fig. 4:**
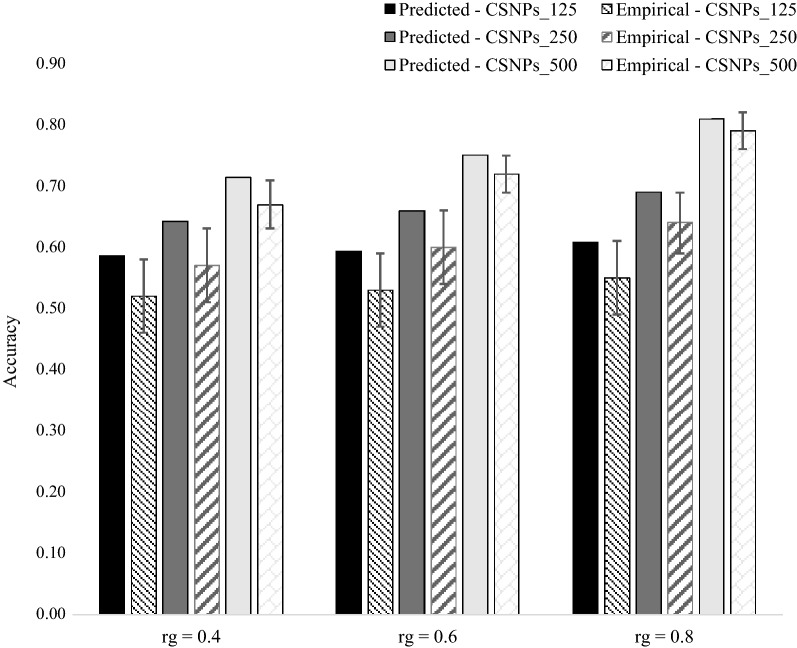
Predicted versus empirical accuracies of the multi-population, multiple ${\mathbf{GRM}}$ genomic prediction model (5553 individuals from population $\varvec{B}$ and 476 individuals from population $\varvec{A}$ in the training population) under the three different levels of pre-selection of causal SNPs, with genetic correlation ($\varvec{r}_{\varvec{g}}$) between populations $\varvec{A}$ and $\varvec{B}$ of 0.4, 0.6 and 0.8, and a heritability of 0.3 in both populations. The standard error bars represent twice the standard deviation of the accuracies across 100 replicates

The empirical and predicted accuracies of the different levels of pre-selection of causal SNPs, with a simulated genetic correlation between populations $A$ and $B$ of 0.4, 0.6 and 0.8 and a heritability of 0.3 in both populations, are in Fig. 4.

Results show that empirical and predicted accuracies increase with an increasing number of pre-selected causal SNPs in the first ${\mathbf{GRM}}$, and the level of genetic correlation between populations. Using the prediction equation (Eq. 17), predicted accuracies were less than one standard error away from the average empirical accuracy in seven of the nine scenarios evaluated. We observed over-prediction of accuracies in all the scenarios, but only in the CSNP_125 and the CSNP_250 and with a genetic correlation between populations of 0.4 did the predicted accuracies go outside the standard error of the empirical accuracies.

**Fig. 5:**
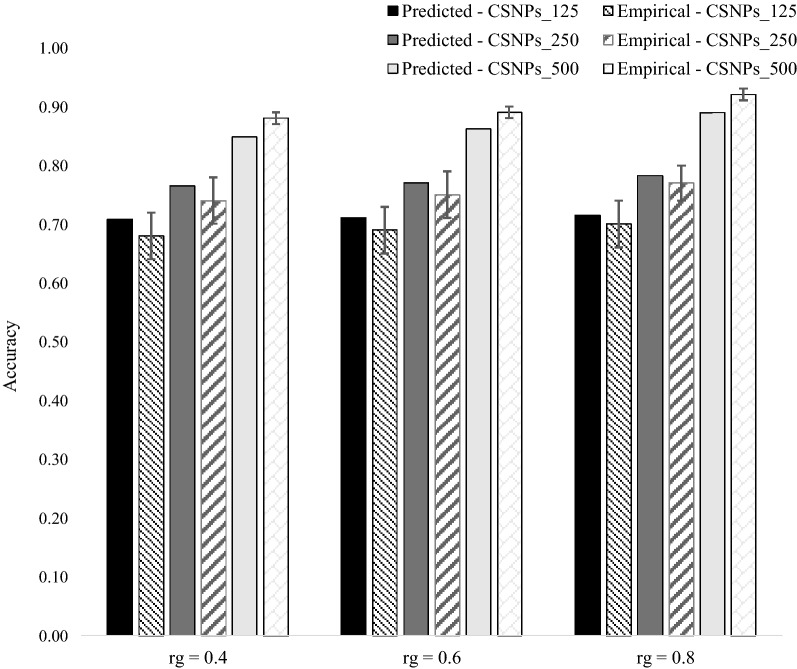
Predicted versus empirical accuracies of the multi-population, multiple ${\mathbf{GRM}}$ genomic prediction model (5553 individuals from population $\varvec{B}$ and 476 individuals from population $\varvec{A}$ in the training population) under the three different levels of pre-selection of causal SNPs, with genetic correlation ($\varvec{r}_{\varvec{g}}$) between populations $\varvec{A}$ and $\varvec{B}$ of 0.4, 0.6 and 0.8, and a heritability of 0.8 in both populations. The standard error bars represent twice the standard deviation of the accuracies across 100 replicates

For a higher heritability trait (0.8), similar patterns of results were observed (Fig. 5). Empirical and predicted accuracies increase with increasing number of pre-selected causal SNPs in the first ${\mathbf{GRM}}$, and the level of genetic correlation between populations. We also observed slight over-predictions of accuracy in the CSNP_125 and CSNP_250 levels, across the three levels of genetic correlation between populations. However, for the CSNP_500 level, we observed a slight under-prediction of accuracies, across the three levels of genetic correlation between populations. The level of over-estimation of empirical accuracy seems to be consistently higher at low heritability (Fig. 4) than at high heritability (Fig. 5). This also the case for the WPMG model (Fig. 1) and for most scenarios of the MPSG model (Figs. 2 and 3). A possible explanation might be that for a lower heritability trait, the SNPs have more difficulty in explaining all the genetic variance. This can result from a larger environmental effect in the phenotypes, which can be considered as a noise term in the phenotype around the genetic effect. Furthermore, the level of over-estimation of empirical accuracies with the MPMG model (between ~ 2 and ~ 10%, Figs. 4 and 5) is within the range of over-estimation observed with the MPSG model (between ~ 5 and 10%, Figs. 2 and 3). Hence, the relative advantage of the MPMG model over the MPSG model as assessed by the corresponding deterministic prediction equations should be a good indication of the true advantage of the MPMG over the MPSG model.

In general, we observed a positive correlation between the EGV from the two ${\mathbf{GRM}}$ in MPMG, albeit with high standard errors, except in the CSNP_500 for which all the QTL underlying the trait are in one ${\mathbf{GRM}}$, where the correlation was around zero (see Additional file 1: Table S1).

### Potential accuracies of different models in relation to different levels of $\varvec{r}_{\varvec{g}}$, $\varvec{ \rho }_{{\varvec{A}_{1} }}$, and $\varvec{M}_{{\varvec{e}_{{\varvec{AB}_{2} }} }}$

**Fig. 6:**
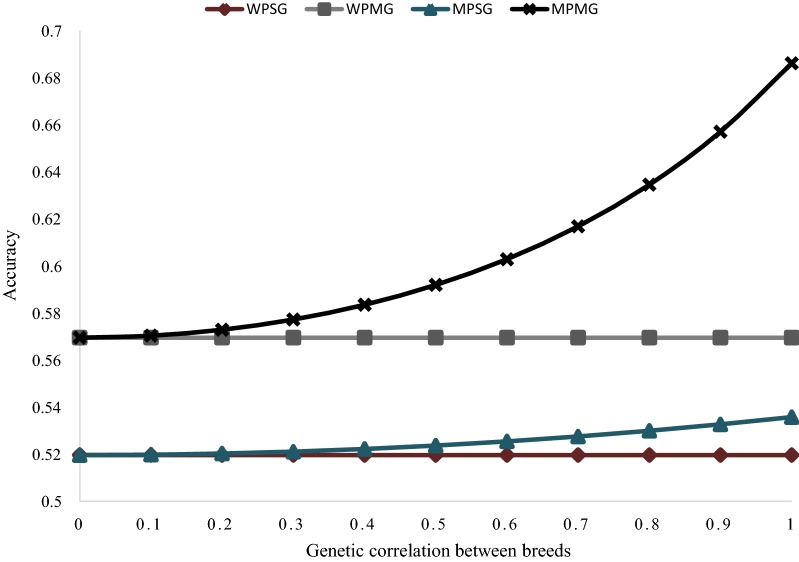
Potential accuracy of predicting the genomic value of individuals from population A under different models: within-population, single ${\mathbf{GRM}}$ (WPSG), within-population, multiple ${\mathbf{GRM}}$ (WPMG), multi-population, single ${\mathbf{GRM}}$ (MPSG), multi-population, multiple ${\mathbf{GRM}}$ (MPMG), in relation to different values of genetic correlation ($\varvec{r}_{\varvec{g}}$) between population $\varvec{A}$ and $\varvec{B}$. The following assumptions were made: $M_{e}$ within population A based on 500 pre-selected causal SNPs (calculated from real genotype data) = 159; $M_{e}$ across populations $A$ and $B$ based on 500 pre-selected causal SNPs (calculated from real genotype data) = 463; $M_{e}$ within population $A$ based on 48,412 non- causal SNPs (calculated from real genotype data) = 280; $M_{e}$ across populations $A$ and $B$ based on 48,412 non-causal SNPs (calculated from real genotype data) = 32,970; $M_{e}$ within population $A$ based on all 48,912 SNPs (calculated from real genotype data) = 280; $M_{e}$ across populations $A$ and $B$ based on all 48,912 SNPs (calculated from real genotype data) = 33,242; heritability of the trait = 0.3 in both populations; proportion of genetic variance explained by all SNPs = 0.8; proportion of genetic variance explained by 500 pre-selected causal SNPs = 0.4; number of individuals from population $A$ in the training population = 476; number of individuals from population $B$ in the training population = 5553

The potential accuracies of within-and multi-population GP models, with either single or multiple ${\mathbf{GRM}}$ fitted, in relation to different levels of genetic correlation ($r_{g}$; case 1) between populations are presented in Fig. 6. The accuracy of the within-population, single ${\mathbf{GRM}}$ (WPSG) model, is not affected by $r_{g}$ between populations. The result shows that a 9.6% increase in accuracy is possible by splitting SNPs into separate ${\mathbf{GRM}}$ based on prior information on their causality (WPMG model), when the preselected SNPs explain 40% of the total genetic variance for the trait. Compared to WPSG, the multi-population, single ${\mathbf{GRM}}$ (MPSG) model can result in a small increase in the accuracy of prediction, ranging from 0% ($r_{g}$ = 0) to a maximum of 3.1% ($r_{g}$ = 1). When the multi-population, multiple ${\mathbf{GRM}}$ (MPMG) model is implemented, the increase in accuracy as compared to the WPSG model ranges from 9.6% ($r_{g}$ = 0) to 32% ($r_{g}$ = 1), again, assuming that the preselected SNPs explain 40% of the genetic variance.

**Fig. 7:**
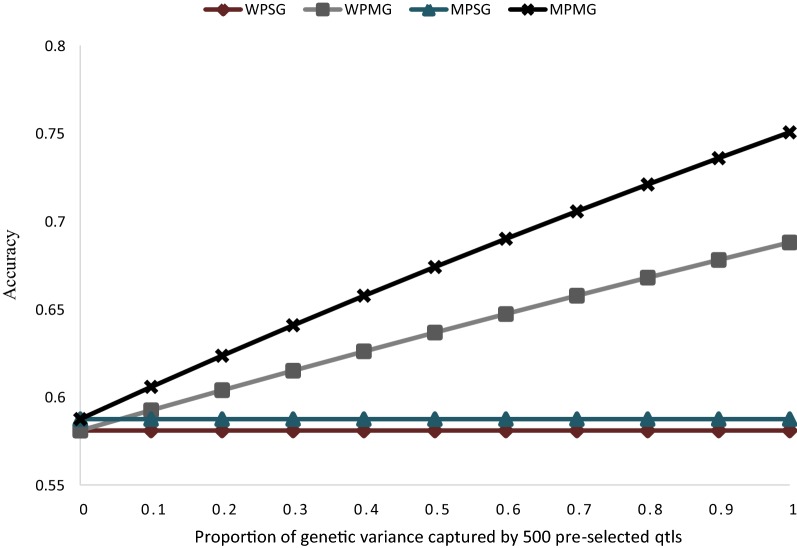
Potential accuracy of predicting the genomic value of individuals from population A under different models: within-population, single ${\mathbf{GRM}}$ (WPSG), within-population, multiple ${\mathbf{GRM}}$ (WPMG), multi-population, single ${\mathbf{GRM}}$ (MPSG), multi-population, multiple ${\mathbf{GRM}}$ (MPMG), in relation to different proportion of genetic variance explained by the causal SNP set. The following assumptions were made: $M_{e}$ within population $A$ based on 500 pre-selected causal SNPs (calculated from real genotype data) = 159; $M_{e}$ across populations $A$ and $B$ based on 500 pre-selected causal SNPs (calculated from real genotype data) = 463; $M_{e}$ within population $A$ based on 48,412 non- causal SNPs (calculated from real genotype data) = 280; $M_{e}$ across populations $A$ and $B$ based on 48,412 non-causal SNPs (calculated from real genotype data) = 32,970; $M_{e}$ within population $A$ based on all 48,912 SNPs (calculated from real genotype data) = 280; $M_{e}$ across populations $A$ and $B$ based on all 48,912 SNPs (calculated from real genotype data) = 33,242; heritability of the trait = 0.3 in both populations; genetic correlation between populations $A$ and $B$ = 0.6; proportion of genetic variance explained by all SNPs = 0.8; number of individuals from population $A$ in the training population = 476; number of individuals from population $B$ in the training population = 5553

The potential accuracies of the different models in relation to different proportions of total genetic variance explained by the pre-selected SNP set ($\rho_{{A_{1} }}$; case 2) are presented in Fig. 7. When the pre-selected SNP set explains zero proportion of genetic variance, the accuracy is equal for the models with one or two ${\mathbf{GRM}}$ for both within- and multi-population GP. Simply implementing the multi-population single ${\mathbf{GRM}}$ model, instead of the within-population single ${\mathbf{GRM}}$ model results in a negligible (< 1%) increase in the accuracy of prediction. An increasing proportion of genetic variance explained by the pre-selected SNP set results in a linear increase in the accuracy of prediction, up to a maximum of ~ 18.4% when the WPMG model is implemented instead of the WPSG model. Increase in accuracy, as compared to the WPSG model, reaches a maximum of ~ 29.2% when the MPMG model is implemented instead.

**Fig. 8:**
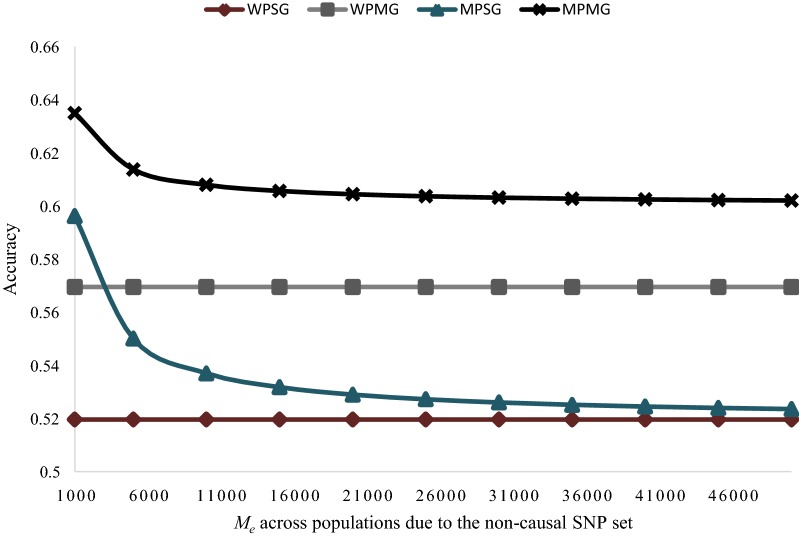
Potential accuracy of predicting the genomic value of individuals from population $\varvec{A}$ under different models: within-population, single ${\mathbf{GRM}}$ (WPSG), within-population, multiple ${\mathbf{GRM}}$ (WPMG), multi-population, single ${\mathbf{GRM}}$ (MPSG), multi-population, multiple ${\mathbf{GRM}}$ (MPMG), in relation to different values of $\varvec{M}_{\varvec{e}}$ across populations due to the non-causal SNP set. The following assumptions were made: $M_{e}$ within population $A$ based on 500 pre-selected causal SNPs (calculated from real genotype data) = 159; $M_{e}$ across populations $A$ and $B$ based on 500 pre-selected causal SNPs (calculated from real genotype data) = 463; $M_{e}$ within population $A$ based on 48,412 non- causal SNPs (calculated from real genotype data) = 280; $M_{e}$ within population $A$ based on all 48,912 SNPs (calculated from real genotype data) = 280; heritability of the trait = 0.3 in both populations; genetic correlation between populations $A$ and $B$ = 0.6; proportion of genetic variance explained by all SNPs = 0.8; proportion of genetic variance explained by 500 pre-selected causal SNPs = 0.4; number of individuals from population $A$ in the training population = 476; number of individuals from population $B$ in the training population = 5553

We investigated further the impact of the number of independent chromosome segments between populations based on the non-causal SNPs ($M_{{e_{AB} }}$; case 3) on the accuracy of GP models, the results are presented in Fig. 8. For the WPSG and WPMG models, the parameter $M_{{e_{AB} }}$ has no relevance. However, in both the MPSG and MPMG models, the accuracy of prediction decreases with increasing $M_{{e_{AB} }}$. The rate of decrease in accuracy with increasing $M_{{e_{AB} }}$, however, is higher in the MPSG model than in the MPMG model. For example, an increase in $M_{{e_{AB} }}$ from 1000 to 20,000 resulted in an 11.3% decrease in accuracy of the MPSG model, while this decrease was only 4.8% in the MPMG model. In general, the difference in accuracy between the MPSG and MPMG models increases with increasing $M_{{e_{AB} }}$. For $M_{{e_{AB} }}$ values smaller than 5000, both multi-population models (MPSG and MPMG) have higher accuracies than any within-population GP model. For larger values of $M_{{e_{AB} }}$, however, the accuracy of the MPSG model is lower than that of the WBMG model, while the accuracy of the MPMG model tends to flatten above that of the WPMG model.

Furthermore, we assessed the potential bias in the predicted accuracies due to the under-estimation of $M_{e}$ within the predicted population $A$. Across different GP models, an underestimation of $M_{e}$ within population $A$ of ~ 20% resulted in an inflation of predicted accuracies ranging between 4.5 (MPMG model) to 7.4% (WPSG model). At level of under-estimation of 90%, inflation of predicted accuracies ranged from 36 (MPMG) to 58% (WPSG)”.

## Discussion

The objective of this study was to underpin theoretically the advantages and limits of the multi-population, multiple ${\mathbf{GRM}}$ (MPMG) genomic prediction model over the multi-population, single ${\mathbf{GRM}}$ (MPSG) genomic prediction (GP) model, by deriving and validating a deterministic prediction equation for its accuracy. We derived the deterministic prediction equation for the accuracy of the MPMG model using selection index theory and building upon previous works by Daetwyler et al. [6] and Wientjes et al. [16]. We showed that, the derived equation can predict the accuracy of the MPMG model under varying levels of genetic correlation between the target population and the additional population in the training population, varying levels of the heritability of the trait and varying levels of the proportions of genetic variance explained by the pre-selected and differentially weighted SNPs. The equation can be used to assess the potential benefit of combining information from different populations, e.g., different breeds or lines for GP in livestock or plants, or different groups of people based on their ethnic background for the prediction of disease risk scores.

To date, in the literature, increase in the accuracy of GP in a multi-population, single-${\mathbf{GRM}}$ (MPSG) context as compared to within-population GP has been limited as illustrated in Table 2 of the review paper of Lund et al. [32]. This is consistent with our result shown in Fig. 7, where less than 1% increase in accuracy is projected if the MPSG model is implemented, assuming a genetic correlation of 0.6 between populations, instead of a within-population, single-${\mathbf{GRM}}$ model. However, Raymond et al. [20] showed that the MPMG model, which differentially weights SNPs based on prior knowledge of potential causality, can yield significant increases in the accuracy of GP as compared to a within-population or multi-population model in which all markers are equally weighted. The prediction equation developed in this study, highlights two parameters: number of independent chromosome segments across populations $A$ and ($M_{{e_{AB} }}$), and the proportion of genetic variance explained by pre-selected SNPs ($\rho_{{A_{1} }}^{2}$), that may underlie the improved performance of the MPMG model as compared to a single ${\mathbf{GRM}}$ model. These parameters and their estimation are discussed below. We also discuss the values for the genetic correlation between populations as used in the prediction equation.

The number of independent chromosome segments across populations ($M_{{e_{AB} }}$) is an important parameter that influences the accuracy of multi-population GP, since it determines the effective number of effects that are estimated in the model [22, 33]. For example, Wientjes et al. [16] showed that when $M_{{e_{AB} }}$ is large, combining populations together in a multi-population single ${\mathbf{GRM}}$ model is less likely to result in a significant increase in accuracy as compared to single-population GP model. However, the prediction equation developed here shows that the MPMG model is still able to take advantage of information from distantly related populations (large $M_{{e_{AB} }}$), mainly by partitioning the parameter $M_{{e_{AB} }}$ into two components, corresponding to pre-selected and remaining SNPs, respectively. In most cases, the value of $M_{{e_{AB} }}$ due to the pre-selected SNPs is small, given that, in most cases, only a few hundred SNPs are pre-selected from e.g., a genome-wide association study. The small value for $M_{{e_{AB} }}$ due to pre-selected SNPs means that the accuracy contributed by the pre-selected SNPs is high, and completely unaffected by the value for $M_{{e_{AB} }}$ due to the remaining SNPs.

For distantly related populations such as Holstein and Jersey, a good estimate for $M_{{e_{AB} }}$ based on a few pre-selected SNPs is the number of pre-selected SNPs (Table 1), which in any case is the maximum possible value. For $M_{{e_{AB} }}$ based on the remaining SNPs, it is very difficult to get an estimate without genotype data, as this value depends on the relatedness between populations. Similar to Wientjes et al. [16], our suggestion is to consider genotyping a sample of individuals from each of the populations, e.g. 100 each, from which $M_{{e_{AB} }}$ can be estimated. With the availability of genotype data on individuals from the target population, the number of independent chromosome segments within population ($M_{{e_{A} }}$) can be estimated empirically as the reciprocal of the variance of within-population genomic relationships [27, 28, 34]. It is also possible to estimate $M_{{e_{A} }}$ based on population parameters such as the effective population size [22, 35, 36]. However, this approach cannot be used to partition $M_{{e_{A} }}$ into values corresponding to the pre-selected and remaining SNPs, respectively, which are required for the prediction equation.

**Fig. 9:**
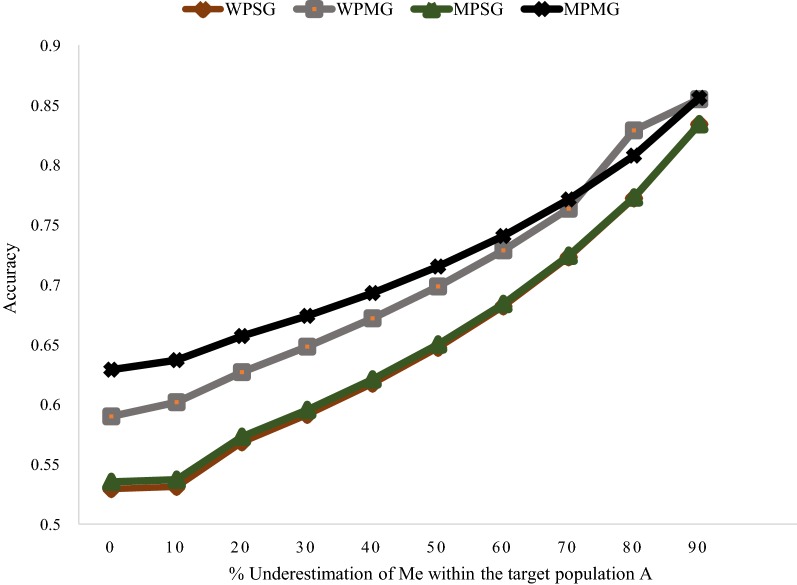
Potential accuracy of predicting the genomic value of individuals from population $\varvec{A}$ under different models: within-population, single ${\mathbf{GRM}}$ (WPSG), within-population, multiple ${\mathbf{GRM}}$ (WPMG), multi-population, single ${\mathbf{GRM}}$ (MPSG), multi-population, multiple ${\mathbf{GRM}}$ (MPMG), in relation to different levels of underestimation of ${\mathbf{M}}_{{\mathbf{e}}}$ within the predicted population $\varvec{A}$ (0 – 90%). The following assumptions were made: ${\text{M}}_{\text{e}}$ across populations $A$ and $B$ based on 500 pre-selected SNPs (calculated from real genotype data) = 280; ${\text{M}}_{\text{e}}$ across populations $A$ and $B$ based on 48,412 remaining SNPs (calculated from real genotype data) = 32,970; genetic correlations between populations $A$ and $B$ = 0.6; heritability of the trait = 0.3 in both populations; proportion of genetic variance explained by SNPs in ${\mathbf{GRM}}_{1}$ = 0.5; number of individuals from population $A$ in the training population = 476; number of individuals from population $B$ in the training population = 5553

All the $M_{e}$ values used in our prediction equation were estimated from the ${\mathbf{GRM}}$. Van den Berg et al. [31] argued that $M_{e}$ values estimated from the ${\mathbf{GRM}}$ are under-estimated, and, when used in a prediction equation, result in the over-prediction of empirical accuracy. We observed that an underestimation of $M_{e}$ within the target population $A$ results in a substantial inflation of predicted accuracies (Fig. 9). The prediction equation developed in our study, with $M_{e}$ values calculated from the ${\mathbf{GRM}}$ tend to over-predict the accuracy of the MPMG model, although in most of the scenarios evaluated, predicted accuracies were still within the standard errors of empirical accuracies. However, the extent of over-prediction of accuracy in van den Berg et al. [31], using the deterministic formula of Goddard et al. [22] with $M_{e}$ values estimated from the ${\mathbf{GRM}}$, was much higher than in our study. We cannot pinpoint, with certainty, the underlying reasons for the differences in the extent of over-prediction of empirical accuracies in our study and in van den Berg et al. [31]. However, there are a few possible explanations. For example, van den Berg et al. [31] fitted a polygenic component (pedigree-based relationship matrix) in their model, which was meant to capture the genetic variance for the trait that is not picked up by SNPs. Although the authors did not present the results of variance components estimation, it is likely that the polygenic component picked up some proportion of the total genetic variance for the trait. If that is the case, using a prediction equation with the assumption that SNPs explain 100% of the total genetic variance for the trait, as the authors did, could have resulted in the over-prediction of empirical accuracies. The chances that the polygenic component picks up some proportion of the total genetic variance for the trait are higher when QTL are sampled from sequence variants that have low MAF, and effects that are difficult to estimate or regressed heavily towards zero in the model, than when QTL are sampled from common SNPs [16]. Furthermore, in their prediction equation, van den Berg et al. [31] corrected for the reduction in prediction error variance as the accuracy of predicted genomic values increases. This correction results in higher predicted accuracies than when the correction is not applied. In our study, however, this correction was not applied. There are other factors, such as the difference in the design of cross-validation schemes to calculate empirical accuracies and structure of the populations analyzed that could potentially underlie the difference in the extent of over-prediction of empirical accuracies between the two studies.

The ability of the MPMG model to take advantage of the small value of $M_{{e_{AB} }}$ due to pre-selected SNPs depends on the accuracy of SNP pre-selection, which in turn determines the proportion of genetic variance explained by the pre-selected SNPs. Our results (Fig. 7) show that for an improvement in accuracy of GP by using the MPMG instead of the MPSG model, it is not sufficient to split randomly SNPs into two classes without accurate prior knowledge on potential causality of the SNPs. Instead, the pre-selected SNPs must explain some proportion of the genetic variance for the trait. Our results agree with those of Sarup et al. [33], who showed that a significant improvement in accuracy by using the “genomic feature” model over the standard GBLUP could be achieved provided the prioritised variants explained more than 10% of the total genetic variance for the trait. In their study, Sarup et al. [33] fitted a linear mixed model including two random genomic effects, with the genetic effects estimated from “genomic feature” or prioritized variants and the remaining variants, respectively. In general, when the pre-selected SNPs explain zero proportion of genetic variance, the accuracy of the MPMG model is expected to be the same as the accuracy of the MPSG model (Fig. 7). Furthermore, if the prior information is accurate, such that the pre-selected SNPs explain some proportion of the genetic variance for the trait, fitting the pre-selected SNPs in a separate ${\mathbf{GRM}}$ in a within-population GP scenario may be more beneficial than combining distantly related populations in a multi-population single-${\mathbf{GRM}}$ model (Fig. 6).

In this study, the proportions of total genetic variance explained in the validation population by the SNPs in the first ${\mathbf{GRM}}$ ($\rho_{{A_{1} }}^{2}$) was determined in a simulation context. There was 100% certainty that the selected SNPs were causal, and that $\rho_{{A_{1} }}^{2}$ did not depend on linkage disequilibrium (LD) between non-causal SNPs and the underlying causal SNPs. In this case, we found that the estimate of genomic heritability (proportion of the genetic variance explained by the SNPs) from the GREML model [37, 38] was equivalent to $\rho_{{A_{1} }}^{2}$(results not shown). de los Campos et al. [39] also showed that when all causal mutations are included in the GREML analysis, the genomic heritability parameter is equivalent to the proportion of genetic variance explained by SNPs ($\rho^{2} )$. However, in practice, the causal mutations that underlie a trait are not always observed, and pre-selected SNPs will usually come from GWAS studies, in which SNPs can show an association with a trait due to LD with unobserved causal mutations. When SNPs that are proxies for causal mutations are used for the analysis, the variance components estimates depend on the effects of the unobserved causal mutations, the extent of LD between SNPs and the causal mutations, and the extent of LD between the SNPs themselves [39]. Not accounting explicitly for the LD between SNPs and the causal mutations in the model used for analysis can result in the over-estimation of variance components [39]. This means that the estimate of genomic heritability from the GREML model cannot be considered to be equivalent to $\rho^{2}$. Browning et al. [40] also showed that population structure can inflate SNP-based heritability estimates. Although genomic heritability from GREML models are higher than $\rho^{2}$, they are still a good indication for $\rho^{2}$, as they have similar trend [41]. Thus, for comparison of the proportion of genetic variance explained by SNPs in different SNP sets, genomic heritability from a GREML model can be used.

In practice, when two ${\mathbf{GRM}}$ are fitted simultaneously in a bivariate GREML model, two estimates of genetic correlation are obtained, one for each ${\mathbf{GRM}}$. However, in this study, we used only the simulated value for the genetic correlation between populations for both ${\mathbf{GRM}}$ in the prediction Eq. (17). This is because the pre-selected SNPs were randomly sampled from all causal SNPs. In practice, a general expectation is that causal SNPs that are pre-selected from e.g. GWAS have a higher effect on the trait than the remaining unselected SNPs, and are most likely to be more consistent across populations [20, 42]. Therefore, the genetic correlation of the preselected SNPs can be higher than that for the remaining SNPs. In the cases that this expectation holds, it is inappropriate to use a single value of genetic correlation for both ${\mathbf{GRM}}$ in the prediction Eq. (17). A solution to this issue is to use the estimated genetic correlation. However, one must take into account that the estimated genetic correlation can be biased when the causal and non-causal SNPs used to estimate the genetic correlation do not have similar properties, e.g., similar pattern of allele frequencies [43]. In general, it is likely that causal SNPs have lower MAF than non-causal SNPs, which means that estimates of genetic correlation obtained by non-causal SNPs can be underestimating the genetic correlation between populations at the causal SNPs [20].

In the derivation of the predicting Eq. (17), we assumed that there is no covariance between ${\mathbf{EGV}}$ from different ${\mathbf{GRM}}$, since the expectation is zero when the ${\mathbf{GRM}}$ are jointly fitted [25]. Because a negative sampling covariance might appear when effects cannot be estimated independently, and the covariance might bias predicted accuracies using the derived equation, we tested this assumption. We calculated the correlations between ${\mathbf{EGV}}_{{{\mathbf{A}}_{1} }}$ and ${\mathbf{EGV}}_{{{\mathbf{A}}_{2} }}$ in each replicate and averaged these across replicates. In general, we observed a positive correlation between the ${\mathbf{EGV}}$ from the two ${\mathbf{GRM}}$ in MPMG, albeit with high standard errors, except in the CSNP_500 in which case all the causal SNPs underlying the trait are in one ${\mathbf{GRM}}$, where the correlation was around 0 (see Additional file 1: Table S1). However, in most cases the correlations were not significantly different from 0. The positive correlation between the ${\mathbf{EGV}}$ mean that individuals that have a high ${\mathbf{EGV}}$ based on the pre-selected SNPs (${\mathbf{GRM}}_{1}$), on average also have a high ${\mathbf{EGV}}$ based on the rest of the genome (${\mathbf{GRM}}_{2}$). To check if the observed correlation between ${\mathbf{EGV}}$ is an artefact of our simulation, we summed the effects of all causal SNPs in ${\mathbf{GRM}}_{1}$ as ${\mathbf{TGV}}_{1}$ and summed the effect of all causal SNPs not in ${\mathbf{GRM}}_{2}$ as ${\mathbf{TGV}}_{2}$. Across 100 replicates, we observed no correlation between the ${\mathbf{TGV}}$: -0.007 (0.07) for CSNP_125 and 0.01 (0.07) for CSNP_250. The observed correlation between ${\mathbf{EGV}}$ do not seem to be a result of sampling covariance, since the expected sign of the resulting correlation is negative, and are not an artefact of our simulation, given that the ${\mathbf{TGV}}$ based on the separate sets of causal SNPs were not correlated. We think that the empirical correlation between ${\mathbf{EGV}}$ are a result of an estimation issue, but we are not sure. In any case, given that the level of overestimation of empirical accuracy of the MPMG model is in the range of overestimation observed in a model with only one $\varvec{GRM}$ (MPSG), the impact of any possible covariance between the ${\mathbf{EGV}}$ on the predicted accuracy of the MPMG model is expected to be small.

## Conclusions

In this paper, we presented a deterministic prediction equation for the accuracy of a multi-population, multiple ${\mathbf{GRM}}$ (MPMG) model, which gives insight into the underlying reasons for the superior performance of the MPMG model over the multi-population, single ${\mathbf{GRM}}$ (MPSG) model. With the help of the prediction equation, we showed that an important advantage of the MPMG model is its ability to benefit from the small number of independent chromosome segments ($M_{e}$) due to the pre-selected SNPs, both within and across populations, while for the MPSG model, there is only a single value for $M_{e}$, calculated based on all SNPs. However, this advantage depends on the condition that the pre-selected SNPs can explain some proportion of the total genetic variance for the trait. The prediction equation developed here can be used as a deterministic tool to assess the potential benefit of combining information from different populations e.g., different breeds or lines for GP in livestock or plants, or different groups of people based on their ethnic background for genomic prediction in humans. This is especially the case, when accurate biological information about the causality of the SNPs is available.

## Supplementary information

**Additional file 1: Table S1.** Estimated correlations between the two estimated genomic values (**EGV**) from the multi-population, multiple genomic relationship matrices (MPMG) model. Standard error of estimates are in parentheses.

## Appendices

### Appendix 1

18 $$\begin{aligned} cov\left( {{\mathbf{EGV}}_{{A_{1} ,A}} ,{\mathbf{EGV}}_{{A_{1} ,B}} } \right) & = cov\left( {\mathop \sum \limits_{j} X_{{A_{i,j} }} \hat{\beta }_{{A_{1j} }} r_{g} \mathop \sum \limits_{j} X_{{A_{i,j} }} \hat{\beta }_{{B_{1j} }} } \right) \hfill \\ & = r_{g} cov\left( {\hat{\beta }_{{A_{1} }} ,\hat{\beta }_{{B_{1} }} } \right), \hfill \\ \hfill \\ \end{aligned}$$

where $X_{{A_{i,j} }}$ is the genotype of validation candidate $i$ from population $A$ at locus $j$, $\hat{\beta }_{{A_{1j} }}$ and $\hat{\beta }_{{B_{1j} }}$ are the estimated SNP effects for locus $j$ in training populations $A$ and $B$, respectively, and $r_{g}$ is the genetic correlation between populations $A$ and $B$.

$$cov\left( {\hat{\beta }_{{A_{1} }} ,\hat{\beta }_{{B_{1} }} } \right) = cor_{{\hat{\beta }_{{A_{1} }} ,\hat{\beta }_{{B_{1} }} }} \sqrt {var\left( {\hat{\beta }_{{A_{1} }} } \right)var\left( {\hat{\beta }_{{B_{1} }} } \right)} ,$$

where $cor_{{\hat{\beta }_{{A_{1} }} ,\hat{\beta }_{{B_{1} }} }}$ is the correlation between the estimated SNP effects of ${\mathbf{GRM}}_{1}$ in populations $A$ and $B$. Using the path coefficient method of Dekkers [23], it can be shown that $cor_{{\hat{\beta }_{{A_{1} }} ,\hat{\beta }_{{B_{1} }} }} = r_{g} r_{{SNP_{{A_{1} }} }} r_{{SNP_{{B_{1} }} }}$, where $r_{{SNP_{{A_{1} }} }}$ and $r_{{SNP_{{B_{1} }} }}$ are the accuracies of the estimated SNP effects in populations $A$ and $B$, respectively. The square root of the variance of estimated SNP effects in populations $A$ and $B$ is equal to the accuracy of the estimated SNP effects, i.e. $\sqrt {{\mathop{\rm var}} \left( {{{\hat \beta }_{{A_{1j}}}}} \right){\mathop{\rm var}} \left( {{{\hat \beta }_{{B_{1j}}}}} \right)} = {r_{SN{P_{A1}}}}{r_{SN{P_{B1}}}}.$.

Therefore:

$$\begin{aligned}cov\left( {\hat{\beta }_{{A_{1} }} ,\hat{\beta }_{{B_{1} }} } \right) & = r_{g} r_{{SNP_{{A_{1} }} }} r_{{SNP_{{B_{1} }} }} r_{{SNP_{{A_{1} }} }} r_{{SNP_{{B_{1} }} }} \\ & = r_{g} r_{{SNP_{{A_{1} }} }}^{2} r_{{SNP_{{B_{1} }} }}^{2} , \end{aligned}$$

and filling this in Eq. (18) results in:

19 $${r_g}{\mathop{\rm cov}} \left( {{{\hat \beta }_{{A_{1j}}}},{{\hat \beta }_{{B_{1j}}}}} \right) = r_g^2r_{SN{P_{{A_1}}}}^2r_{SN{P_{{B_1}}}}^2$$

Assuming that true genomic values are scaled such that they have a variance of 1, Eq. (19) becomes:

20 $$r_{{{\mathbf{EGV}}_{{A_{1} ,A}} }}^{2} r_{{{\mathbf{EGV}}_{{A_{1} ,B}} }}^{2} .$$

### Appendix 2

Matrix ${\mathbf{P}}$ and vector ${\mathbf{g}}$ are shown below:

$${\mathbf{P}} = \left[ {\begin{array}{*{20}c} {r_{{{\mathbf{EGV}}_{{A_{1} ,A}} }}^{2} } \\ {r_{{{\mathbf{EGV}}_{{A_{1} ,A}} }}^{2} r_{{{\mathbf{EGV}}_{{A_{1} ,B}} }}^{2} } \\ 0 \\ 0 \\ \end{array} \begin{array}{*{20}c} {r_{{{\mathbf{EGV}}_{{A_{1} ,A}} }}^{2} r_{{{\mathbf{EGV}}_{{A_{1} ,B}} }}^{2} } \\ {r_{{{\mathbf{EGV}}_{{A_{1} ,B}} }}^{2} } \\ 0 \\ 0 \\ \end{array} \begin{array}{*{20}c} 0 \\ 0 \\ {r_{{{\mathbf{EGV}}_{{A_{2} ,A}} }}^{2} } \\ {r_{{{\mathbf{EGV}}_{{A_{2} ,A}} }}^{2} r_{{{\mathbf{EGV}}_{{A_{2} ,B}} }}^{2} } \\ \end{array} \begin{array}{*{20}c} 0 \\ 0 \\ {r_{{{\mathbf{EGV}}_{{A_{2} ,A}} }}^{2} r_{{{\mathbf{EGV}}_{{A_{2} ,B}} }}^{2} } \\ {r_{{{\mathbf{EGV}}_{{A_{2} ,B}} }}^{2} } \\ \end{array} } \right] .$$

$${\mathbf{g}} = \left[ {\begin{array}{*{20}c} {\begin{array}{*{20}c} {r_{{{\mathbf{EGV}}_{{A_{1} ,A}} }}^{2} } \\ {r_{{{\mathbf{EGV}}_{{A_{1} ,B}} }}^{2} } \\ \end{array} } \\ {\begin{array}{*{20}c} {r_{{{\mathbf{EGV}}_{{A_{2} ,A}} }}^{2} } \\ {r_{{{\mathbf{EGV}}_{{A_{2} ,B}} }}^{2} } \\ \end{array} } \\ \end{array} } \right].$$

Since **P** is a block diagonal matrix, it can be split into 2x2 sub-matrices for ease of inversion:

$${\mathbf{P}}_{11} = \left[ {\begin{array}{*{20}c} {r_{{{\mathbf{EGV}}_{{A_{1} ,A}} }}^{2} } & {r_{{{\mathbf{EGV}}_{{A_{1} ,A}} }}^{2} r_{{{\mathbf{EGV}}_{{A_{1} ,B}} }}^{2} } \\ {r_{{{\mathbf{EGV}}_{{A_{1} ,A}} }}^{2} r_{{{\mathbf{EGV}}_{{A_{1} ,B}} }}^{2} } & {r_{{{\mathbf{EGV}}_{{A_{1} ,B}} }}^{2} } \\ \end{array} } \right],$$

$${\mathbf{P}}_{22} = \left[ {\begin{array}{*{20}c} {r_{{{\mathbf{EGV}}_{{A_{2} ,A}} }}^{2} } & {r_{{{\mathbf{EGV}}_{{A_{2} ,A}} }}^{2} r_{{{\mathbf{EGV}}_{{A_{2} ,B}} }}^{2} } \\ {r_{{{\mathbf{EGV}}_{{A_{2} ,A}} }}^{2} r_{{{\mathbf{EGV}}_{{A_{2} ,B}} }}^{2} } & {r_{{{\mathbf{EGV}}_{{A_{2} ,B}} }}^{2} } \\ \end{array} } \right].$$

Then, $${\mathbf{P}} = \left[ {\begin{array}{*{20}c} {{\mathbf{P}}_{11} } & 0 \\ 0 & {{\mathbf{P}}_{22} } \\ \end{array} } \right]$$ and $${\mathbf{P}}^{ - 1} = \left[ {\begin{array}{*{20}c} {{\mathbf{P}}_{11}^{ - 1} } & 0 \\ 0 & {{\mathbf{P}}_{22}^{ - 1} } \\ \end{array} } \right]$$. The inverse of the two non-zero sub-matrices of ${\mathbf{P}}$ can be calculated as:

$${\mathbf{P}}_{11}^{ - 1} = \frac{1}{{r_{{{\mathbf{EGV}}_{{A_{1} ,A}} }}^{2} r_{{{\mathbf{EGV}}_{{A_{1} ,B}} }}^{2} - r_{{{\mathbf{EGV}}_{{A_{1} ,A}} }}^{4} r_{{{\mathbf{EGV}}_{{A_{1} ,B}} }}^{4} }}\left[ {\begin{array}{*{20}c} {r_{{{\mathbf{EGV}}_{{A_{1} ,B}} }}^{2} } & { - r_{{{\mathbf{EGV}}_{{A_{1} ,A}} }}^{2} r_{{{\mathbf{EGV}}_{{A_{1} ,B}} }}^{2} } \\ { - r_{{{\mathbf{EGV}}_{{A_{1} ,A}} }}^{2} r_{{{\mathbf{EGV}}_{{A_{1} ,B}} }}^{2} } & {r_{{{\mathbf{EGV}}_{{A_{1} ,A}} }}^{2} } \\ \end{array} } \right]$$

$$=\left[\begin{array}{cc}\frac{{r}_{{\widehat{\mathrm{g}}}_{{A}_{1},B}}^{2}}{{r}_{{\mathbf{E}\mathbf{G}\mathbf{V}}_{{A}_{1},A}}^{2}{r}_{{\mathbf{E}\mathbf{G}\mathbf{V}}_{{A}_{1},B}}^{2}- {r}_{{\mathbf{E}\mathbf{G}\mathbf{V}}_{{A}_{1},A}}^{4}{r}_{{\mathbf{E}\mathbf{G}\mathbf{V}}_{{A}_{1},B}}^{4}}& \frac{-{r}_{{\widehat{\mathrm{g}}}_{{A}_{1},A}}^{2}{r}_{{\widehat{\mathrm{g}}}_{{A}_{1},B}}^{2}}{{r}_{{\mathbf{E}\mathbf{G}\mathbf{V}}_{{A}_{1},A}}^{2}{r}_{{\mathbf{E}\mathbf{G}\mathbf{V}}_{{A}_{1},B}}^{2}- {r}_{{\mathbf{E}\mathbf{G}\mathbf{V}}_{{A}_{1},A}}^{4}{r}_{{\mathbf{E}\mathbf{G}\mathbf{V}}_{{A}_{1},B}}^{4}}\\ \frac{-{r}_{{\widehat{\mathrm{g}}}_{{A}_{1},A}}^{2}{r}_{{\widehat{\mathrm{g}}}_{{A}_{1},B}}^{2}}{{r}_{{\mathbf{E}\mathbf{G}\mathbf{V}}_{{A}_{1},A}}^{2}{r}_{{\mathbf{E}\mathbf{G}\mathbf{V}}_{{A}_{1},B}}^{2}- {r}_{{\mathbf{E}\mathbf{G}\mathbf{V}}_{{A}_{1},A}}^{4}{r}_{{\mathbf{E}\mathbf{G}\mathbf{V}}_{{A}_{1},B}}^{4}}& \frac{{r}_{{\widehat{\mathrm{g}}}_{{A}_{1},A}}^{2}}{{r}_{{\mathbf{E}\mathbf{G}\mathbf{V}}_{{A}_{1},A}}^{2}{r}_{{\mathbf{E}\mathbf{G}\mathbf{V}}_{{A}_{1},B}}^{2}- {r}_{{\mathbf{E}\mathbf{G}\mathbf{V}}_{{A}_{1},A}}^{4}{r}_{{\mathbf{E}\mathbf{G}\mathbf{V}}_{{A}_{1},B}}^{4}}\end{array}\right]$$

$$= \left[ {\begin{array}{*{20}c} {\frac{1}{{r_{{{\mathbf{EGV}}_{{A_{1} ,A}} }}^{2} \left( {r_{{{\mathbf{EGV}}_{{A_{1} ,A}} }}^{2} r_{{{\mathbf{EGV}}_{{A_{1} ,B}} }}^{2} } \right)}}} & {\frac{ - 1}{{\left( {1 - r_{{{\mathbf{EGV}}_{{A_{1} ,A}} }}^{2} r_{{{\mathbf{EGV}}_{{A_{1} ,B}} }}^{2} } \right)}}} \\ {\frac{ - 1}{{\left( {1 - r_{{{\mathbf{EGV}}_{{A_{1} ,A}} }}^{2} r_{{{\mathbf{EGV}}_{{A_{1} ,B}} }}^{2} } \right)}}} & {\frac{1}{{r_{{{\mathbf{EGV}}_{{A_{1} ,B}} }}^{2} \left( {1 - r_{{{\mathbf{EGV}}_{{A_{1} ,A}} }}^{2} r_{{{\mathbf{EGV}}_{{A_{1} ,B}} }}^{2} } \right)}}} \\ \end{array} } \right].$$

Similarly:

$${\mathbf{P}}_{22}^{ - 1} = \left[ {\begin{array}{*{20}c} {\frac{1}{{r_{{{\mathbf{EGV}}_{{A_{2} ,A}} }}^{2} \left( {1 - r_{{{\mathbf{EGV}}_{{A_{2} ,A}} }}^{2} r_{{{\mathbf{EGV}}_{{A_{2} ,B}} }}^{2} } \right)}}} & {\frac{ - 1}{{\left( {1 - r_{{{\mathbf{EGV}}_{{A_{2} ,A}} }}^{2} r_{{{\mathbf{EGV}}_{{A_{2} ,B}} }}^{2} } \right)}}} \\ {\frac{ - 1}{{\left( {1 - r_{{{\mathbf{EGV}}_{{A_{2} ,A}} }}^{2} r_{{{\mathbf{EGV}}_{{A_{2} ,B}} }}^{2} } \right)}}} & {\frac{1}{{r_{{{\mathbf{EGV}}_{{A_{2} ,B}} }}^{2} \left( {1 - r_{{{\mathbf{EGV}}_{{A_{2} ,A}} }}^{2} r_{{{\mathbf{EGV}}_{{A_{2} ,B}} }}^{2} } \right)}}} \\ \end{array} } \right].$$

$${r}_{{\mathbf{E}\mathbf{G}\mathbf{V}}_{{A}_{T}}}=\sqrt{{\left[\begin{array}{c}\begin{array}{c}{r}_{{\mathbf{E}\mathbf{G}\mathbf{V}}_{{A}_{1},A}}^{2}\\ {r}_{{\mathbf{E}\mathbf{G}\mathbf{V}}_{{A}_{1},B}}^{2}\end{array}\\ \begin{array}{c}{r}_{{\mathbf{E}\mathbf{G}\mathbf{V}}_{{A}_{2},A}}^{2}\\ {r}_{{\mathbf{E}\mathbf{G}\mathbf{V}}_{{A}_{2},B}}^{2}\end{array}\end{array}\right]}^{T}\left[\begin{array}{c}\begin{array}{c}\frac{1}{{r}_{{\mathbf{E}\mathbf{G}\mathbf{V}}_{{A}_{1},A}}^{2}({r}_{{\mathbf{E}\mathbf{G}\mathbf{V}}_{{A}_{1},A}}^{2}{r}_{{\mathbf{E}\mathbf{G}\mathbf{V}}_{{A}_{1},B}}^{2})}\\ \frac{-1}{(1-{r}_{{\mathbf{E}\mathbf{G}\mathbf{V}}_{{A}_{1},A}}^{2}{r}_{{\mathbf{E}\mathbf{G}\mathbf{V}}_{{A}_{1},B}}^{2})}\end{array}\\ \begin{array}{c}0\\ 0\end{array}\end{array} \begin{array}{c}\begin{array}{c}\frac{-1}{(1-{r}_{{\mathbf{E}\mathbf{G}\mathbf{V}}_{{A}_{1},A}}^{2}{r}_{{\mathbf{E}\mathbf{G}\mathbf{V}}_{{A}_{1},B}}^{2})}\\ \frac{1}{{r}_{{\mathbf{E}\mathbf{G}\mathbf{V}}_{{A}_{1},B}}^{2}(1-{r}_{{\mathbf{E}\mathbf{G}\mathbf{V}}_{{A}_{1},A}}^{2}{r}_{{\mathbf{E}\mathbf{G}\mathbf{V}}_{{A}_{1},B}}^{2})}\end{array}\\ \begin{array}{c}0\\ 0\end{array}\end{array} \begin{array}{c}\begin{array}{c}0\\ 0\end{array}\\ \begin{array}{c}\frac{1}{{r}_{{\mathbf{E}\mathbf{G}\mathbf{V}}_{{A}_{2},A}}^{2}(1-{r}_{{\mathbf{E}\mathbf{G}\mathbf{V}}_{{A}_{2},A}}^{2}{r}_{{\mathbf{E}\mathbf{G}\mathbf{V}}_{{A}_{2},B}}^{2})}\\ \frac{-1}{(1-{r}_{{\mathbf{E}\mathbf{G}\mathbf{V}}_{{A}_{2},A}}^{2}{r}_{{\mathbf{E}\mathbf{G}\mathbf{V}}_{{A}_{2},B}}^{2})}\end{array}\end{array} \begin{array}{c}\begin{array}{c}0\\ 0\end{array}\\ \begin{array}{c}\frac{-1}{(1-{r}_{{\mathbf{E}\mathbf{G}\mathbf{V}}_{{A}_{2},A}}^{2}{r}_{{\mathbf{E}\mathbf{G}\mathbf{V}}_{{A}_{2},B}}^{2})}\\ \frac{1}{{r}_{{\mathbf{E}\mathbf{G}\mathbf{V}}_{{A}_{2},B}}^{2}(1-{r}_{{\mathbf{E}\mathbf{G}\mathbf{V}}_{{A}_{2},A}}^{2}{r}_{{\mathbf{E}\mathbf{G}\mathbf{V}}_{{A}_{2},B}}^{2})}\end{array}\end{array}\right]\left[\begin{array}{c}\begin{array}{c}{r}_{{\mathbf{E}\mathbf{G}\mathbf{V}}_{{A}_{1},A}}^{2}\\ {r}_{{\mathbf{E}\mathbf{G}\mathbf{V}}_{{A}_{1},B}}^{2}\end{array}\\ \begin{array}{c}{r}_{{\mathbf{E}\mathbf{G}\mathbf{V}}_{{A}_{2},A}}^{2}\\ {r}_{{\mathbf{E}\mathbf{G}\mathbf{V}}_{{A}_{2},B}}^{2}\end{array}\end{array}\right] }$$

Simplifying the equation above results in:

21 $$\sqrt{\begin{array}{c}\frac{{r}_{{\mathbf{E}\mathbf{G}\mathbf{V}}_{{A}_{1},A}}^{2}}{\left(1-{r}_{{\mathbf{E}\mathbf{G}\mathbf{V}}_{{A}_{1},A}}^{2}{r}_{{\mathbf{E}\mathbf{G}\mathbf{V}}_{{A}_{1},B}}^{2}\right)}+\frac{-{r}_{{\mathbf{E}\mathbf{G}\mathbf{V}}_{{A}_{1},A}}^{2}{r}_{{\mathbf{E}\mathbf{G}\mathbf{V}}_{{A}_{1},B}}^{2}}{\left(1-{r}_{{\mathbf{E}\mathbf{G}\mathbf{V}}_{{A}_{1},A}}^{2}{r}_{{\mathbf{E}\mathbf{G}\mathbf{V}}_{{A}_{1},B}}^{2}\right)}+\frac{-{r}_{{\mathbf{E}\mathbf{G}\mathbf{V}}_{{A}_{1},A}}^{2}{r}_{{\mathbf{E}\mathbf{G}\mathbf{V}}_{{A}_{1},B}}^{2}}{\left(1-{r}_{{\mathbf{E}\mathbf{G}\mathbf{V}}_{{A}_{1},A}}^{2}{r}_{{\mathbf{E}\mathbf{G}\mathbf{V}}_{{A}_{1},B}}^{2}\right)}+\frac{{r}_{{\mathbf{E}\mathbf{G}\mathbf{V}}_{{A}_{1},B}}^{2}}{\left(1-{r}_{{\mathbf{E}\mathbf{G}\mathbf{V}}_{{A}_{1},A}}^{2}{r}_{{\mathbf{E}\mathbf{G}\mathbf{V}}_{{A}_{1},B}}^{2}\right)}\\ +\frac{{r}_{{\mathbf{E}\mathbf{G}\mathbf{V}}_{{A}_{2},A}}^{2}}{(1-{r}_{{\mathbf{E}\mathbf{G}\mathbf{V}}_{{A}_{2},A}}^{2}{r}_{{\mathbf{E}\mathbf{G}\mathbf{V}}_{{A}_{2},B}}^{2})}+\frac{-{r}_{{\mathbf{E}\mathbf{G}\mathbf{V}}_{{A}_{2},A}}^{2}{r}_{{\mathbf{E}\mathbf{G}\mathbf{V}}_{{A}_{2},B}}^{2}}{(1-{r}_{{\mathbf{E}\mathbf{G}\mathbf{V}}_{{A}_{2},A}}^{2}{r}_{{\mathbf{E}\mathbf{G}\mathbf{V}}_{{A}_{2},B}}^{2})}+\frac{-{r}_{{\mathbf{E}\mathbf{G}\mathbf{V}}_{{A}_{2},A}}^{2}{r}_{{\mathbf{E}\mathbf{G}\mathbf{V}}_{{A}_{2},B}}^{2}}{(1-{r}_{{\mathbf{E}\mathbf{G}\mathbf{V}}_{{A}_{2},A}}^{2}{r}_{{\mathbf{E}\mathbf{G}\mathbf{V}}_{{A}_{2},B}}^{2})}+\frac{{r}_{{\mathbf{E}\mathbf{G}\mathbf{V}}_{{A}_{2},B}}^{2}}{(1-{r}_{{\mathbf{E}\mathbf{G}\mathbf{V}}_{{A}_{2},A}}^{2}{r}_{{\mathbf{E}\mathbf{G}\mathbf{V}}_{{A}_{2},B}}^{2})}\end{array}}$$

The reliability of estimated genomic values is a product of the reliability of the estimated SNP effects ($$r_{SNP}^{2} )$$ and the proportion of genetic variance explained in the validation population by the SNPs ($$\rho^{2} )$$. Ignoring $$\rho^{2}$$, for now, and replacing $$r_{SNP}^{2}$$ with the deterministic equations to predict accuracy of GP [6, 27]:

$$r_{{SNP_{{A_{1} }} }}^{2} = \sqrt {\frac{{h_{A}^{2} N_{A} }}{{h_{A}^{2} N_{A} + ME_{{A_{1} }} }}} = \sqrt {\frac{{\frac{{h_{A}^{2} }}{{ME_{{A_{1} }} }}}}{{\frac{{h_{A}^{2} }}{{ME_{{A_{1} }} }} + \frac{1}{{N_{A} }}}}} ,$$

$$r_{{SNP_{{B_{1} }} }}^{2} = \sqrt {r_{g}^{2} \frac{{\frac{{h_{A}^{2} }}{{ME_{{AB_{1} }} }}}}{{\frac{{h_{B}^{2} }}{{ME_{{AB_{1} }} }} + \frac{1}{{N_{B} }}}}} ,$$

$$r_{{SNP_{{A_{2} }} }}^{2} = \sqrt {\frac{{\frac{{h_{A}^{2} }}{{ME_{{A_{2} }} }}}}{{\frac{{h_{A}^{2} }}{{ME_{{A_{2} }} }} + \frac{1}{{N_{A} }}}}} ,$$

$${\text{and}}\;\;r_{{SNP_{{B_{2} }} }}^{2} = \sqrt {r_{g}^{2} \frac{{\frac{{h_{A}^{2} }}{{ME_{{AB_{2} }} }}}}{{\frac{{h_{B}^{2} }}{{ME_{{AB_{2} }} }} + \frac{1}{{N_{B} }}}}} ,$$

then Eq. (21) becomes:

22 $$\sqrt{\begin{array}{c}\left(\frac{\frac{\frac{{h}_{A}^{2}}{{ME}_{{A}_{1}}}}{\frac{{h}_{A}^{2}}{{ME}_{{A}_{1}}}+\frac{1}{{N}_{A}}}+{r}_{g}^{2}\frac{\frac{{h}_{A}^{2}}{{ME}_{{AB}_{1}}}}{\frac{{h}_{B}^{2}}{{ME}_{{AB}_{1}}}+\frac{1}{{N}_{B}}}-2\left(\frac{\frac{{h}_{A}^{2}}{{ME}_{{A}_{1}}}}{\frac{{h}_{A}^{2}}{{ME}_{{A}_{1}}}+\frac{1}{{N}_{A}}}\right)\left({r}_{g}^{2}\frac{\frac{{h}_{A}^{2}}{{ME}_{{AB}_{1}}}}{\frac{{h}_{B}^{2}}{{ME}_{{AB}_{1}}}+\frac{1}{{N}_{B}}}\right)}{1-\left(\frac{\frac{{h}_{A}^{2}}{{ME}_{{A}_{1}}}}{\frac{{h}_{A}^{2}}{{ME}_{{A}_{1}}}+\frac{1}{{N}_{A}}}\right)\left({r}_{g}^{2}\frac{\frac{{h}_{A}^{2}}{{ME}_{{AB}_{1}}}}{\frac{{h}_{B}^{2}}{{ME}_{{AB}_{1}}}+\frac{1}{{N}_{B}}}\right)}\right)\\ +\left(\frac{\frac{\frac{{h}_{A}^{2}}{{ME}_{{A}_{2}}}}{\frac{{h}_{A}^{2}}{{ME}_{{A}_{2}}}+\frac{1}{{N}_{A}}}+{r}_{g}^{2}\frac{\frac{{h}_{A}^{2}}{{ME}_{{AB}_{2}}}}{\frac{{h}_{B}^{2}}{{ME}_{{AB}_{2}}}+\frac{1}{{N}_{B}}}-2\left(\frac{\frac{{h}_{A}^{2}}{{ME}_{{A}_{2}}}}{\frac{{h}_{A}^{2}}{{ME}_{{A}_{2}}}+\frac{1}{{N}_{A}}}\right)\left({r}_{g}^{2}\frac{\frac{{h}_{A}^{2}}{{ME}_{{AB}_{2}}}}{\frac{{h}_{B}^{2}}{{ME}_{{AB}_{2}}}+\frac{1}{{N}_{B}}}\right)}{1-\left(\frac{\frac{{h}_{A}^{2}}{{ME}_{{A}_{2}}}}{\frac{{h}_{A}^{2}}{{ME}_{{A}_{2}}}+\frac{1}{{N}_{A}}}\right)\left({r}_{g}^{2}\frac{\frac{{h}_{A}^{2}}{{ME}_{{AB}_{2}}}}{\frac{{h}_{B}^{2}}{{ME}_{{AB}_{2}}}+\frac{1}{{N}_{B}}}\right)}\right)\end{array}}$$

Multiplying both the numerator and the denominator of the first component of Eq. (22) by $\left( {\frac{{h_{A}^{2} }}{{ME_{{A_{1} }} }} + \frac{1}{{N_{A} }}} \right)\left( {\frac{{h_{B}^{2} }}{{ME_{{AB_{1} }} }} + \frac{1}{{N_{B} }}} \right)$ and the second component by $\left( {\frac{{h_{A}^{2} }}{{ME_{{A_{2} }} }} + \frac{1}{{N_{A} }}} \right)\left( {\frac{{h_{A}^{2} }}{{ME_{{A_{2} }} }} + \frac{1}{{N_{A} }}} \right)$ results in:

23 $$\sqrt{\begin{array}{c}\left(\frac{\frac{{h}_{A}^{2}}{{ME}_{{A}_{1}}}\left(\frac{{h}_{B}^{2}}{{ME}_{{AB}_{1}}}+\frac{1}{{N}_{B}}\right)+{r}_{g}^{2}\frac{{h}_{B}^{2}}{{ME}_{{AB}_{1}}}\left(\frac{{h}_{A}^{2}}{{ME}_{{A}_{1}}}+\frac{1}{{N}_{A}}\right)-2\left(\frac{{h}_{A}^{2}}{{ME}_{{A}_{1}}}\right)\left({r}_{g}^{2}\frac{{h}_{B}^{2}}{{ME}_{{AB}_{1}}}\right)}{\left(\frac{{h}_{A}^{2}}{{ME}_{{A}_{1}}}+\frac{1}{{N}_{A}}\right)\left(\frac{{h}_{B}^{2}}{{ME}_{{AB}_{1}}}+\frac{1}{{N}_{B}}\right)-\left(\frac{{h}_{A}^{2}}{{ME}_{{A}_{1}}}\right)\left({r}_{g}^{2}\frac{{h}_{B}^{2}}{{ME}_{{AB}_{1}}}\right)}\right)\\ + \left(\frac{\frac{{h}_{A}^{2}}{{ME}_{{A}_{2}}}\left(\frac{{h}_{B}^{2}}{{ME}_{{AB}_{2}}}+\frac{1}{{N}_{B}}\right)+{r}_{g}^{2}\frac{{h}_{B}^{2}}{{ME}_{{AB}_{2}}}\left(\frac{{h}_{A}^{2}}{{ME}_{{A}_{2}}}+\frac{1}{{N}_{A}}\right)-2\left(\frac{{h}_{A}^{2}}{{ME}_{{A}_{2}}}\right)\left({r}_{g}^{2}\frac{{h}_{B}^{2}}{{ME}_{{AB}_{2}}}\right)}{\left(\frac{{h}_{A}^{2}}{{ME}_{{A}_{2}}}+\frac{1}{{N}_{A}}\right)\left(\frac{{h}_{B}^{2}}{{ME}_{{AB}_{2}}}+\frac{1}{{N}_{B}}\right)-\left(\frac{{h}_{A}^{2}}{{ME}_{{A}_{2}}}\right)\left({r}_{g}^{2}\frac{{h}_{B}^{2}}{{ME}_{{AB}_{2}}}\right)}\right)\end{array}}$$

Taking into account the proportion of genetic variance explained by SNPs in ${\mathbf{GRM}}_{1}$ ($\rho_{{A_{1} }}^{2}$) and ${\mathbf{GRM}}_{2}$ ($\rho_{{A_{2} }}^{2}$), respectively, Eq. (23) becomes:

24 $$\sqrt{\begin{array}{c}{\rho }_{{A}_{1}}^{2}\left(\frac{\frac{{h}_{A}^{2}}{{ME}_{{A}_{1}}}\left(\frac{{h}_{B}^{2}}{{ME}_{{AB}_{1}}}+\frac{1}{{N}_{B}}\right)+{r}_{g}^{2}\frac{{h}_{B}^{2}}{{ME}_{{AB}_{1}}}\left(\frac{{h}_{A}^{2}}{{ME}_{{A}_{1}}}+\frac{1}{{N}_{A}}\right)-2\left(\frac{{h}_{A}^{2}}{{ME}_{{A}_{1}}}\right)\left({r}_{g}^{2}\frac{{h}_{B}^{2}}{{ME}_{{AB}_{1}}}\right)}{\left(\frac{{h}_{A}^{2}}{{ME}_{{A}_{1}}}+\frac{1}{{N}_{A}}\right)\left(\frac{{h}_{B}^{2}}{{ME}_{{AB}_{1}}}+\frac{1}{{N}_{B}}\right)-\left(\frac{{h}_{A}^{2}}{{ME}_{{A}_{1}}}\right)\left({r}_{g}^{2}\frac{{h}_{B}^{2}}{{ME}_{{AB}_{1}}}\right)}\right)\\ + {\rho }_{{A}_{2}}^{2}\left(\frac{\frac{{h}_{A}^{2}}{{ME}_{{A}_{2}}}\left(\frac{{h}_{B}^{2}}{{ME}_{{AB}_{2}}}+\frac{1}{{N}_{B}}\right)+{r}_{g}^{2}\frac{{h}_{B}^{2}}{{ME}_{{AB}_{2}}}\left(\frac{{h}_{A}^{2}}{{ME}_{{A}_{2}}}+\frac{1}{{N}_{A}}\right)-2\left(\frac{{h}_{A}^{2}}{{ME}_{{A}_{2}}}\right)\left({r}_{g}^{2}\frac{{h}_{B}^{2}}{{ME}_{{AB}_{2}}}\right)}{\left(\frac{{h}_{A}^{2}}{{ME}_{{A}_{2}}}+\frac{1}{{N}_{A}}\right)\left(\frac{{h}_{B}^{2}}{{ME}_{{AB}_{2}}}+\frac{1}{{N}_{B}}\right)-\left(\frac{{h}_{A}^{2}}{{ME}_{{A}_{2}}}\right)\left({r}_{g}^{2}\frac{{h}_{B}^{2}}{{ME}_{{AB}_{2}}}\right)}\right)\end{array}}$$
