# Supplementary material for: A deterministic equation to predict the accuracy of multi-population genomic prediction with multiple genomic relationship matrices
Source: Genet Sel Evol. 2020 Apr 28;52:21. doi: 10.1186/s12711-020-00540-y (PMC7189707; doi:10.1186/s12711-020-00540-y)
Supplement: Supplementary file 1 — Additional file 1: Table S1. Estimated correlations between the two estimated genomic values (EGV) from the multi-population, multiple genomic relationship matrices (MPMG) model. Standard error of estimates are in parentheses. [file 12711_2020_540_MOESM1_ESM.docx]

**Table S1**: Estimated correlations between the 2 estimated genomic values (EGVs) from the multi-population, multiple genomic relationship matrices (MPMG) model. Standard error of estimates are in parenthesis.

| GRM 1* | Correlation (EGV1, EGV2), (h^2= 0.3) | Correlation (EGV1, EGV2) (h^2 = 0.8) |
| --- | --- | --- |
| $r_{g}$ = 0.4 | | |
| CSNPs_125 | 0.22 (0.14) | 0.19 (0.08) |
| CSNPs_250 | 0.30 (0.16) | 0.26 (0.08) |
| CSNPs_500 | -0.04 (0.40) | 0.03 (0.28) |
| $r_{g}$ = 0.6 | | |
| CSNPs_125 | 0.18 (0.13) | 0.16 (0.09) |
| CSNPs_250 | 0.23 (0.15) | 0.22 (0.09) |
| CSNPs_500 | -0.07 (0.34) | 0.03 (0.25) |
| $r_{g}$ = 0.8 | | |
| CSNPs_125 | 0.13 (0.12) | 0.11 (0.09) |
| CSNPs_250 | 0.14 (0.15) | 0.16 (0.09) |
| CSNPs_500 | -0.07 (0.27) | 0.02 (0.20) |

**GRM 2 throughout the study was made from ~48k non-causal SNPs.*
